# Hepatic targeting of the centrally active cannabinoid 1 receptor (CB_1_R) blocker rimonabant via PLGA nanoparticles for treating fatty liver disease and diabetes

**DOI:** 10.1016/j.jconrel.2022.11.040

**Published:** 2022-11-30

**Authors:** Shira Hirsch, Liad Hinden, Meital Ben-David Naim, Saja Baraghithy, Anna Permyakova, Shahar Azar, Taher Nasser, Emma Portnoy, Majd Agbaria, Alina Nemirovski, Gershon Golomb, Joseph Tam

**Affiliations:** a Obesity and Metabolism Laboratory, POB 12065, Jerusalem 9112001, Israel; b The Institute for Drug Research, School of Pharmacy, Faculty of Medicine, The Hebrew University of Jerusalem, Israel; c Department of Biochemistry, Institute for Medical Research Israel-Canada, Hebrew University-Hadassah Medical School, Israel

**Keywords:** CB1 receptor blocker, Obesity, Hepatic steatosis, Insulin resistance, Drug delivery system, Polymeric nanoparticles, Nanomedicine

## Abstract

Over-activation of the endocannabinoid/CB1R system is a hallmark feature of obesity and its related comorbidities, most notably type 2 diabetes (T2D), and non-alcoholic fatty liver disease (NAFLD). Although the use of drugs that widely block the CB1R was found to be highly effective in treating all metabolic abnormalities associated with obesity, they are no longer considered a valid therapeutic option due to their adverse neuro-psychiatric side effects. Here, we describe a novel nanotechnology-based drug delivery system for repurposing the abandoned first-in-class global CB1R antagonist, rimonabant, by encapsulating it in polymeric nanoparticles (NPs) for effective hepatic targeting of CB1Rs, enabling effective treatment of NAFLD and T2D. Rimonabant-encapsulated NPs (Rimo-NPs) were mainly distributed in the liver, spleen, and kidney, and only negligible marginal levels of rimonabant were found in the brain of mice treated by iv/ip administration. In contrast to freely administered rimonabant treatment, no CNS-mediated behavioral activities were detected in animals treated with Rimo-NPs. Chronic treatment of diet-induced obese mice with Rimo-NPs resulted in reduced hepatic steatosis and liver injury as well as enhanced insulin sensitivity, which were associated with enhanced cellular uptake of the formulation into hepatocytes. Collectively, we successfully developed a method of encapsulating the centrally acting CB_1_R blocker in NPs with desired physicochemical properties. This novel drug delivery system allows hepatic targeting of rimonabant to restore the metabolic advantages of blocking CB_1_R in peripheral tissues, especially in the liver, without the negative CB_1_R-mediated neuropsychiatric side effects.

## 1 Introduction

Non-alcoholic fatty liver disease (NAFLD), a common and potentially serious condition often associated with obesity, is a major cause of morbidity and mortality [1]. It is characterized by a spectrum of liver conditions, ranging from an ectopic accumulation of fat in the liver (hepatic steatosis), to non-alcoholic steatohepatitis (NASH), which can be complicated by fibrosis, cirrhosis, end-stage liver failure, and hepatocellular carcinoma (HCC). Several lines of evidence suggest that NAFLD promotes type 2 diabetes (T2D). Although NAFLD is present in 20–30% of the general population [2], it reaches the impressive prevalence of 50–75% in patients affected by T2D [3]. Once T2D is fully developed, it further contributes not only to the development of steatosis but also to NASH, fibrosis, cirrhosis, and possibly HCC [4]. Therefore, early therapeutic interventions are imperative for the treatment of NAFLD patients at risk for developing T2D.

Recent findings from our lab and from others have revealed the significant role played by the endocannabinoid (eCB) system in the pathogenesis of T2D and NAFLD. eCBs are endogenous lipid ligands that interact with the cannabinoid receptors, CB1R and CB2R, which also recognize Δ^9^-tetrahydrocannabinol (THC), the psychoactive component of marijuana, and mediate its biological effects [5,6]. By activating CB_1_Rs, eCBs increase the appetite (the ‘munchies’) and lipogenesis in adipose tissue and liver [7,8] as well as induce insulin resistance and dyslipidemia [9,10]. In addition, elevated circulating levels of eCBs have been reported in obese patients vs. lean controls; they are positively associated with waist circumference, body mass index (BMI), visceral adiposity, insulin resistance, and NAFLD [11–16]. Thus, an overactive eCB/CB_1_R system contributes to the development of visceral obesity, hepatic steatosis, T2D, and other medical complications [17].

Consequently, pharmaceutical companies have been encouraged to develop drugs that block CB_1_Rs as a potential treatment for these clinical conditions. The first such compound, rimonabant (SR141716, Acomplia®, Sanofi-Aventis), was found effective, not only in reducing body weight in obese and overweight individuals, but also in ameliorating the associated metabolic abnormalities, including hepatic steatosis, insulin resistance, and T2D [18–23]. However, the neuropsychiatric side effects, including depression, anxiety, and suicidal ideation, led to its worldwide withdrawal as a viable medicine in 2009, and halted further therapeutic development of this class of compounds [24]. In addition, preclinical evidence has emerged indicating that CB_1_R in peripheral tissues is mostly involved in hormonal and metabolic regulation [25]. This raised the prospect that selective targeting of peripheral CB_1_R may retain some or most of its metabolic benefits while avoiding any neuropsychiatric liability. In addition, we have recently provided comprehensive evidence for the therapeutic potential of targeting the peripheral CB_1_R for the treatment of obesity, T2D, NAFLD, chronic kidney disease, and osteoporosis [26–34].

In view of the pivotal role of CB_1_R in the pathogenesis of NAFLD and T2D, targeting the disease-activated CB_1_Rs in the liver is an attractive strategy to stop or reverse these pathologies. Thus, nanomedicine provides a feasible approach to target centrally acting insoluble lipophilic drugs, such as rimonabant, directly to the liver while reducing its centrally mediated adverse effects. To test this hypothesis, we formulated PLGA ((poly(lactic-*co*-glycolic acid))-encapsulated rimonabant nano-particles (Rimo-NPs) and demonstrated that they effectively inhibit hepatic CB_1_R and improve obesity-induced hepatic steatosis and insulin resistance without any apparent neuropsychiatric side effects.

## 2 Materials & methods

### 2.1 Reagents

Rimonabant hydrochloride was purchased from Glentham (Cat #GP8358). PLGA, which has a lactide-glycolide ratio of 50:50 and a MW of 30,000–60,000 (acid-terminated), was purchased from Lactel Absorbable Polymers (Cat #B6013–2). PLGA-Cyanine 5 endcap, which has a lactide-glycolide ratio of 50:50 and a MW of 30,000–55,000 (acid-terminated), was purchased from PolySciTech (Cat #AV034). Poly(vinyl alcohol) (PVA, 30–70 kDa) was purchased from Sigma-Aldrich (Cat #P8136). Phosphate salts were purchased from Biological Industries (Cat #11–223-1G). AM251 was purchased from Cayman Chemical Company (Cat #71670).

### 2.2 Nanoparticles preparation

Various formulation attempts have been experimented (Supplementary Information). The selected Rimo-NP formulation was prepared by the emulsion evaporation technique. Briefly, a solution of 10 mg of rimonabant in 200 μL EtOH was added to 3 mL of dichloromethane (DCM) containing 3% PLGA. The solution was added dropwise to a 2% PVA solution (in 25 mL 1xPBS buffer); then, it was sonicated for 105 s at 80% amplitude over an ice bath to form a single emulsion (O/W). DCM was eliminated by evaporation under reduced pressure using a rotary evaporator (Buchi, Switzerland), which resulted in NP formation. The NPs were recovered by ultracentrifugation (20,000 rpm for 20 min at 4 °C), then washed twice (with a 10% albumin solution, and finally with 1xPBS) to remove PVA and unentrapped rimonabant. Next, the pellet was resuspended in a 10% sucrose solution and lyophilized. Dry lyophilized NPs were stored at −20 °C until use. Drug-free NPs (empty NPs) were prepared by the same procedure, except that rimonabant was omitted. Fluorescent NPs were prepared by replacing 10% of the PLGA with Cyanine-5-labeled PLGA.

### 2.3 Physicochemical characterization of NPs

#### 2.3.1 Size, surface charge, and morphology

NP size and surface charge (*Z* potential) were determined by dynamic light scattering (DLS) at room temperature (Zetasizer Nano-ZSP, Malvern Instruments, UK). An aliquot (50 μL) of the NP suspension, obtained before lyophilization or after resuspending the lyophilized NPs in 1xPBS buffer, was added to 1 mL HPLC water. NP morphology was assessed utilizing transmission electron microscopy (TEM, JEM-1400 Plus, JEOL Ltd., Tokyo, Japan). Briefly, 5 μL samples were stained with NanoVan™ (Nanoprobes, NY, USA) and then placed on formvar/carbon-coated copper 200 mesh grids (EMS), and mixed with 5 μL of NV for 5–10 s. Excess stain was blotted off and grids were dried. The grids were viewed with Jeol® JEM-1400 Plus TEM (Jeol®, Tokyo, Japan). The grids were equipped with an ORIUS SC600 CCD camera (Gatan®, Abingdon, United Kingdom); the Gatan Microscopy Suite program was used (DigitalMicrograph, Gatan®, UK).

#### 2.3.2 Rimonabant loading efficiency

For each batch, 1–2 mg of NPs was dissolved in 0.1 mL of HPLC water and 0.9 mL of acetonitrile. Samples were centrifuged at 13,000 rpm for 10 min at room temperature; then, the supernatant was analyzed by HPLC (Dionex UltiMate 3000, Thermo Scientific); the equipment consisted of an UltiMate 3000 SD pump and a UV–Vis Absorbance (DAD, MWD, VWD). The separation was performed on a Phenomenex C18 column (4.6 mm × 150 mm) at 225 nm. Next, the mobile phase was prepared with acetonitrile and water (90:10), and was pumped at a rate of 0.6 mL/min at 25 °C. The injection volume for analysis was 10 μL. The concentration of rimonabant was calculated against an appropriate calibration curve. Rimonabant’s loading and encapsulation efficiencies (%) were calculated using the following equations:

$$Rimonabant’s\;loading\;(\text{\%})=\frac{\text{Rimonabant}\;\text{weight}\;(\text{mg})*\times 100}{\text{NP}\;\text{weight}\;(\text{mg})}$$

$$Encapsulation\;efficiency\;(\text{\%})=\frac{\text{Actual}\;\text{rimonabant}\;\text{loading}\;(\text{mg}/\text{mg})\times 100}{\text{Theoretical}\;\text{rimonabant}\;\text{loading}\;{\left(\text{mg}/\text{mg}\right)}^{**}}$$

*Calculated based on the spectrophotometric assay.

**Calculated based on the initial amount of rimonabant used for NP preparation.

#### 2.3.3 Rimonabant-loaded NP stability

To examine the stability of the formulation, which was stored at −20 °C, the size and content of lyophilized NPs were examined at different time points over a period of 2 years by DLS and HPLC.

#### 2.3.4 In vitro drug release

The drug release from rimonabant-loaded NPs was carried out by ultracentrifugation of the NPs and by measuring the amount of rimonabant in the supernatant. Briefly, 0.1 mL of rimonabant-loaded NPs was added into 0.9 mL of 50% human serum in 1xPBS at 4 °C and 37 °C. Samples were collected at specific time points, centrifuged at 45,000 rpm for 75 min at 4 °C; then the drug content in the supernatant and residue was measured using an HPLC.

### 2.4 Cell cultures

Primary mouse hepatocytes were isolated by liberase perfusion as previously described [35]. Briefly, the liver of anesthetized mice was first perfused with calcium-free Hanks balanced salt solution supplemented with HEPES and EDTA (HBSS, Biological Industries, Beit HaE-mek, Cat# 02–018-1A HEPES Cat# 03–025-1B), followed by perfused liberase digestion (25 μg/mL liberase in HBSS; Biological Industries, Beit HaEmek, Cat# 02–015-1A with 25 mM HEPES). After digestion, the hepatocytes were released by dissociation from the lobes (centrifuged at 50 *×g* for 2 min at 4 °C). The pellet from the first centrifugation of hepatocytes was loaded on a 90% Percoll gradient (Sigma-Aldrich; Cat# GE17–0891-01) and centrifuged at 200 *×g* for 10 min at 4 °C. The cells were then cultured on 6-well plates at a density of 4 × 10^5^ cells/well with planting medium (DMEM, Biological Industries, Beit HaEmek, Cat# 01–050-1A) supplemented with 100 U/mL penicillin-streptomycin and 5% FBS, which, was changed after 3 h to William’s E medium (Rhenium; Cat# 22551022) containing 2 mM L-glutamine and 100 U/ mL penicillin-streptomycin. Cells were incubated overnight at 37 °C in a humidified 5%CO_2_/95% air atmosphere.

### 2.5 In vitro cytotoxicity

The cytotoxicity of the rimonabant-loaded NPs was examined in comparison with empty NPs and a free drug solution. Primary hepatocytes were seeded in 12-well plates (200,000 cells/well) containing the complete growth medium. The cells were left to properly adhere for 1 day, and then incubated with different solutions for 24 and 48 h. Cell viability was determined by XTT assay PMS (*N*-methyl dibenzopyrazine methyl sulfate). The plates were quantified by a plate reader at a wavelength of 450–630 nm. Cell viability was normalized to vehicle-treated cells.

### 2.6 Cellular uptake of the NPs

Primary hepatocytes were seeded on coverslips in 12-well plates (200,000 cells/well) containing a complete growth medium and were left overnight to adhere. Cells were incubated for 1, 3, 6, and 24 h with 4.4 mg/mL fluorescent NPs (Cy5-PLGA NPs). The nucleus was stained with DAPI. Next, the cells were washed three times with 1xPBS, fixed using 4% formaldehyde solution for 10 min, washed again with 1xPBS, and mounted onto a microscope slide. Slides were analyzed using a Nikon A1R Confocal laser scanning microscope (magnification of ×60). Cells treated with unlabeled NPs were used as controls.

### 2.7 Animals and the experimental protocol

The experimental protocol used was approved by the Institutional Animal Care and Use Committee of the Hebrew University, which is an AAALAC International accredited institute. To generate diet-induced obesity (DIO; body weight > 42 g), six-week-old male C57Bl/6JHsd mice (Envigo, Israel) were fed either a high-fat diet (HFD; 60% of calories from fat, 20% from protein, and 20% from carbohydrates; Research Diet, D12492), or a standard diet (STD; 14% fat, 24% protein, 62% carbohydrates; NIH-31 rodent diet) for 16 weeks. To assess the metabolic effect of the formulations, the mice were treated ip with Rimo-NPs, a free solution of rimonabant (1 mg/kg, each), or empty NPs (vehicle) for 4 weeks. Body-weight and food intake were measured daily. Total body fat and lean masses were determined by EchoM-RI100H™ (Echo Medical Systems LLC, Houston, TX, USA). Urine was collected one week before euthanasia for a 24 h period using mouse metabolic cages (CCS2000 Chiller System, Hatteras Instruments, NC, USA). Mice were euthanized by cervical dislocation under anesthesia, and the kidneys, brain, liver, fat pads, spleen, lungs, and heart were removed and weighed, and samples were either snap-frozen or fixed in buffered 4% formalin. Trunk blood was collected to determine the biochemical parameters.

### 2.8 Blood and liver biochemistry

Serum levels of cholesterol, triglycerides, high-density lipoprotein (HDL), low-density lipoprotein (LDL), and alanine aminotransferase (ALT) were determined by using the Cobas C-111 chemistry analyzer (Roche, Switzerland). Serum insulin and leptin levels were measured by ELISAs (Merck, Cat #EZRMI-13 K and #E6082-K, respectively). Fasting blood glucose was measured using a glucometer. Liver tissues were extracted as described previously [31], and their triglyceride content was determined using the Cobas C-111 chemistry analyzer (Roche, Switzerland).

### 2.9 Glucose tolerance test (ipGTT) and insulin sensitivity test (ipIST)

Mice that underwent overnight fasting were injected with glucose (1.5 g/kg, ip), followed by a tail blood collection at 0, 15, 30, 45, 60, 90, and 120 min. The blood glucose levels were determined using a glucometer. The following day, the mice underwent fasting for 6 h before receiving insulin (0.75 U/kg, ip; Eli Lilly); their blood glucose levels were determined at the same intervals as above. The homeostasis model assessment insulin resistance (HOMA-IR) was calculated as (insulin [μU/mL] X fasting plasma glucose [mmol/L] / 22.5). The insulin sensitivity index (ISI) was calculated as 1/(glucose x insulin) X 1000, with glucose expressed as mg/dL and insulin as mU/L.

### 2.10 Histopathological analyses

Five μm paraffin-embedded liver sections from 7 animals per group were stained with H&E staining. Liver images were captured with a Zeiss AxioCam ICc5 colour camera mounted on a Zeiss Axio Scope.A1 light microscope and taken from 10 random 40× fields of each animal. Lipid staining was conducted using an Oil Red O staining kit (Cat #ab150678; Abcam) according to the manufacturer’s protocol. Briefly, at a 10 mm optimal cutting temperature (OCT) medium (Bar-Naor, Cat# BN62550), compound-embedded liver sections were placed in propylene glycerol, followed by an Oil Red O solution and hematoxylin staining. Stained sections were photographed as mentioned above and positive red areas were quantified in a blinded manner using Image J software with a minimum of 10 random liver sections per mouse.

### 2.11 Biodistribution of rimonabant-loaded NPs

The biodistribution of Rimo-NPs, in comparison with free rimonabant, was determined in two cohorts of mice: The first cohort included male 10–11-week-old C57bl/6JHsd mice (Envigo, Israel) injected with Rimo-NPs or free rimonabant (iv: 0.01, 0.1, 1, 3 mg/kg; ip: 1 mg/kg). The NPs were suspended in 1xPBS (15–450 μg/mL), and the free drug was dissolved in 4% DMSO, 1% Tween 80, and saline (0.01–3.0 mg/mL). The mice were euthanized 1-, 4-, and 24-h post-injection. The second cohort included HFD-fed obese mice treated chronically (28 days) with Rimo-NPs or free rimonabant (1 mg/kg, ip). The mice were euthanized 18 h following the last injection. Blood was collected, and the brain, lungs, liver, kidneys, fat, and spleen were harvested.

Tissues and serum samples were processed as follows: Total proteins were precipitated with an ice-cold acetone/Tris buffer (50 mM, pH 8.0). Then, the samples were homogenized in ice-cold methanol/Tris buffer (50 mM, pH 8.0, 1:1) containing 10 μg/mL AM251 as an internal standard. Homogenates were then extracted with CHCl_3_:MeOH (2:1, vol/vol) and washed two times with ice-cold CHCl_3_, dried under nitrogen flow, and reconstituted with MeOH. Rimonabant levels in the samples were determined by liquid chromatography/tandem mass spectrometry (LC-MS/MS).

The analyses were conducted on a TSQ Quantum Access Max triple quadrupole mass spectrometer (Thermo Scientific, San Jose, CA, USA) coupled with an UHPLC system, which included a Dionex Pump and an Accela Autosampler (Thermo Scientific, San Jose, CA, USA). A Kinetex (Phenomenex, Torrance, CA, USA) column (C18, 2.7 μm particle size, 50 × 2.1 mm) was used for separation. Gradient elution mobile phases consisted of 0.1% formic acid in water (phase A) and 0.1% formic acid in acetonitrile (phase B). Gradient elution (0.3 mL/min) was held at 40% B for 0.7 min, followed by a linear increase to 85% B for 6.3 min, followed by a linear increase to 95% B for 0.2 min, and it was maintained at 95% B for 3.3 min. Then, it decreased linearly to 40% B for 0.5 min, and it was maintained at 40% B for 6.0 min. Rimonabant was detected in a positive ion mode using electron spray ionization (ESI) and the multiple reaction monitoring (MRM) mode of acquisition. The mass spectrometer parameters were set as follows: spray voltage 4500 V; vaporizer temperature 370 °C; capillary temperature 260 °C; sheath and auxiliary gases 35 and 30 arbitrary units, respectively; argon was used as the collision gas.

The molecular ions and fragments for each compound were measured as follows: *m/z* 463/363 (quantifier), m/z 463/300 (qualifier) for rimonabant (collision energy: 29 V and 40 V, respectively), and m/z 555/454.9 (quantifier) and m/z 555/327.9 (qualifier) for AM251 (collision energy: 30 V and 49 V, respectively). TSQ Tune Software (Thermo Scientific, San Jose, CA, USA) was used to optimize the tuning parameters. Data acquisition and processing were carried out using the Xcalibur program (Thermo Scientific, San Jose, CA, USA). The amounts of rimonabant in the samples were determined against a standard curve. Values are expressed as ng/g or ng/mL in wet tissue weight or serum volume, respectively.

### 2.12 Biodistribution of Cy-5 PLGA NPs

Sixteen-week-old male C57Bl/6JHsd mice were injected ip with fluorescent NPs (PLGA covalently linked to cyanine 5). The NPs were suspended in 1xPBS (21.4 mg/mL), and 200 μL (140 mg/kg NPs) were injected per mouse. The mice were euthanized 1-, 4-, 8-, and 24-h (*n* = 3 in each group and 1 mouse as a control) post-injection and perfused with 1xPBS (via the left ventricle until the effluent from the right atrium was clear of blood). Finally, the brain, lungs, liver, kidneys, fat pads, and spleen were harvested. The accumulation of the fluorescent NPs in the organs was assessed by fluorescent imaging (Typhoon FLA 9500 bio-molecular imager, GE Healthcare, UK), followed by image analysis (ImageJ). The mean fluorescent intensity in each organ was subtracted from that of an animal that was treated with empty NPs as a control. Immediately after the livers were scanned, they were embedded in OCT (Bar-Naor, Cat# BN62550), followed by snap freezing in dry ice; they were stored at −80 °C until cryo-sectioned by using the Leica CM1950 cryostat (Leica Biosystems, Germany).

### 2.13 Immunostaining

Liver sections were prepared as described above and frozen without a mounting medium. Following thawing at room temperature, the sections were washed three times for 5 min each in 1×PBS, fixed (4% formaldehyde) for 15 min, washed three times for 5 min each in 1×PBS, and blocked with 10% Normal Goat Serum in 1×PBS/0.05% Tween-20 for 1 h (for F4/80 staining) or blocked with 10% Normal Goat Serum, 1% BSA, 1% Triton (for albumin staining). Then, the sections were washed three times for 5 min each in 1 × PBS. Kupffer cells were stained overnight with a rat anti-mouse F4/80 antibody (1:100 abcam, Cat# 6640) and hepatocytes were stained overnight with FITC-anti mouse albumin antibody (1:10, Santa Cruz, Cat #271605) at 4 °C. Slides were then washed three times for 10 min each in 1 × PBS (for albumin) or in 1×PBS/0.05% Tween-20 (for F4/80) staining, and incubated with a goat anti-rat FITC-conjugated antibody (1:100, abcam, Cat# ab150157) for 1 h. Following three washing steps of 10 min each, the sections were embedded in Vectashield mounting media containing DAPI (abcam, Cat# ab104139), and the images were captured with a Nikon A1R Confocal laser scanning microscope (magnification of ×60).

### 2.14 Flow cytometry analysis

Six 10–12-week-old male C57Bl/6 J mice (JAX, Bar Harbour) were injected ip with fluorescent NPs (PLGA covalently linked to Cyanine 5). The NPs were suspended in 1xPBS (20.2 mg/mL), and 200 μL (140 mg/ kg NPs) were injected per mouse. The mice (*n* = 3 in each group) were euthanized 1 h post-injection; then hepatocytes and non-parenchymal cells (NPCs) were isolated as described previously [36]. Briefly, mice were anesthetized and perfused with the Liver Perfusion Buffer (Gibco, Cat# 17701–038); then, they were washed in a digestion buffer (HBSS Biological Industries, Beit HaEmek, Cat# 02–018-1A) containing 5 mM CaCl_2_, 10 mM HEPES, and 25 μg/mL collagenase I & II (Liberase TM, Roche Cat# 5401119001). After the digestion procedure, the liver was excised and maintained in the Hepatocyte Wash Media (Gibco, Cat#17704024) on ice. Hepatocytes were released from liver lobes and then filtered through 70-μm cell strainers (SPL Life Sciences, Cat#93070). After centrifugation and washing steps, hepatocytes were re-suspended in 40% Percoll (Sigma; Cat# GE17–0891-01) in Williams E Media (Gibco, Cat# 12551032), and centrifuged (60 ×*g* for 4 min) to collect live hepatocytes on the bottom of the vial. The remaining liver lobes were minced and further digested by using 2.5 mg/mL collagenase D (Roche, Cat# 11088858001) and 100 ng/mL DNAse I (Roche, Cat# 11284932001) at 37 °C for 30 min. After the centrifugation and washing steps, the NPC pellets were loaded on top of Percoll gradient layers (10 mL 15% Percoll on the top and 10 mL 40% Percoll on the bottom) for further centrifugation. The NPC layer in the middle was collected. Isolated hepatocytes and NPCs were first incubated with Fc-receptor blocking, anti-mouse CD16/CD32 antibody (BioLegend, #101301), then stained with FITC anti-mouse CD45 (BioLegend, #157608). Flow cytometry was performed using a CytoFLEX Flow Cytometer (Beckman Coulter, Switzerland). Unstained hepatocytes and NPCs, isolated from mice injected with unlabeled NPs, were used as negative controls.

### 2.15 Real-time qPCR

Total RNA of mouse livers or primary hepatocytes was extracted using TRIzol (Invitrogen), followed by DNase I treatment (Invitrogen), and then reverse-transcribed using an Iscript cDNA kit (Bio-Rad). Real-time PCR was conducted using iTaq Universal SYBR Green Supermix (Bio-Rad) and the CFX connect ST system (Bio-Rad). The list of mouse primers is presented in Supplementary Table 9. The following *mus musculus* genes were detected: *Cpta1*, *Acadm*, *Echs1*, *Hadh*, and *Acaa2*; all genes were normalized to *β-actin*

### 2.16 CB1R antagonism with free rimonabant/rimonabant-NPs

Primary hepatocytes were cultured in M199 medium (Biological Industries, Beit HaEmek, Cat# 01–080-1A) containing 1% fatty acid-free BSA. Free rimonabant or Rimo-NPs were added to the medium to a final concentration of 1 μM, followed by the addition of a mixed solution of sodium oleate (Sigma-Aldrich; Cat# O7501) and sodium palmitate (Sigma-Aldrich; Cat# P9767) at a ratio of 2:1, to a final concentration of 0.5 mM, for 24 h. Then, the cells were harvested in Trizol for qPCR analysis.

### 2.17 Multi-parameter metabolic assessment

The metabolic and activity profiles of the mice were assessed using the Promethion High-Definition Behavioral Phenotyping System (Sable Instruments, Inc., Las Vegas, NV, USA). Data acquisition and instrument control were performed using MetaScreen software version 2.2.18.0, and the obtained raw data were processed using ExpeData version 1.8.4, using an analysis script detailing all aspects of the data transformation. Mice with free access to food and water were subjected to a standard 12 h light/12 h dark cycle, which consisted of a 16 h acclimation period, followed by 24 h of sampling. Respiratory gases were measured by using the GA-3 gas analyzer (Sable Systems, Inc., Las Vegas, NV, USA), using a pull-mode, negative-pressure system. Airflow was measured and controlled by FR-8 (Sable Systems, Inc., Las Vegas, NV, USA), with a set flow rate of 2000 mL/min. Water vapor was continuously measured and its dilution effect on O_2_ and CO_2_ was mathematically compensated. The effective mass (eff.Mass) was calculated by ANCOVA analysis. The respiratory quotient (RQ) was calculated as the ratio between CO_2_ produced to O_2_ consumed, and the total energy expenditure (TEE) was calculated as *VO_2_ x (3.815* + *1.232 x RQ)*, normalized to the effective body mass, and expressed as kcal/h/kg^eff.Mass^. Fat oxidation (FO) was calculated as FO = 1.69 x VO_2_–1.69 x VCO_2_ and expressed as g/d/kg^eff. Mass^. Ambulatory activity and position were monitored simultaneously with the collection of the calorimetry data using XYZ beam arrays with a beam spacing of 0.25 cm.

### 2.18 Elevated plus-maze

Anxiety-related behaviors were assessed using the EPM test, as reported previously [37]. Animals were placed on the 5 × 5 cm central platform of an apparatus from which four arms, 30 cm × 5 cm, extended. Two of the arms (the closed arms) are enclosed within 15 cm high walls, and the two other arms (the open arms) have 1 cm high rims. The whole maze is elevated 75 cm above the ground. During the 6 min test time, the number of entries to each arm type (closed or open arms and frequencies) and the time spent in each type of arm (closed or open arms and durations) during the test were measured.

### 2.19 Catalepsy test

Catalepsy was assayed using the bar test. Briefly, mice were removed from their home cages, and their forepaws were placed on a horizontal bar, 0.5 cm in diameter, positioned 4 cm above the bench surface. Vehicle-treated mice routinely let go of the bar within 2 s. Cataleptic behavior was defined as the time the animals remained motionless holding onto the bar, with an arbitrary cutoff of 30 s. The antagonists (free rimonabant at 1 and 10 mg/kg, Rimo-NPs at 1 mg/kg) were given 30 min before the ip injection of 3 mg/kg WIN55,212. The test was performed 60 min after agonist administration.

### 2.20 Activity profile

Locomotor activity was quantified by the number of disruptions of the infrared beams in 2 dimensions in the Promethion High-Definition Behavioral Phenotyping System (Sable Instruments, Inc., Las Vegas, NV, USA).

### 2.21 Acute food and water intakes

Precise measurements of food and water intakes in male 10–12-week-old C57Bl/6JHsd mice were assessed by using the Promethion High-Definition Behavioral Phenotyping System. Briefly, mice with free access to water were fasted from food for 24 h. The mice were injected with the test drugs 30 min before they were refed. Cumulative food and water intakes were measured for 3 h.

### 2.22 Statistics

Values are expressed as the mean ± SEM. An unpaired two-tailed Student’s *t*-test was used to determine differences between the Veh- and the drug-treated groups. The results in multiple groups and time-dependent variables were compared by ANOVA, followed by a Tukey’s multiple comparisons test (GraphPad Prism v6 for Windows). Significance was at *p* < 0.05.

## 3 Results

### 3.1 Rimo-NPs formulation and characterization

To successfully encapsulate rimonabant in NPs or liposomes, we used various polymers (acid- or ester-terminated PLGA or PLA, having different MWs, and various lactic acid:glycolic acid ratios), diverse nano preparations (nanoprecipitation, single, or double emulsion evaporation), as well as different surfactants (Solutol or PVA). Unfortunately, neither of these methods resulted in efficient entrapment of rimonabant in the nano-drug delivery systems nor prevented its aggregation (For more information, please see the Supplementary Text, Supplementary Table 1–8 and Supplementary Figs. 1–9).

**Fig. 1:**
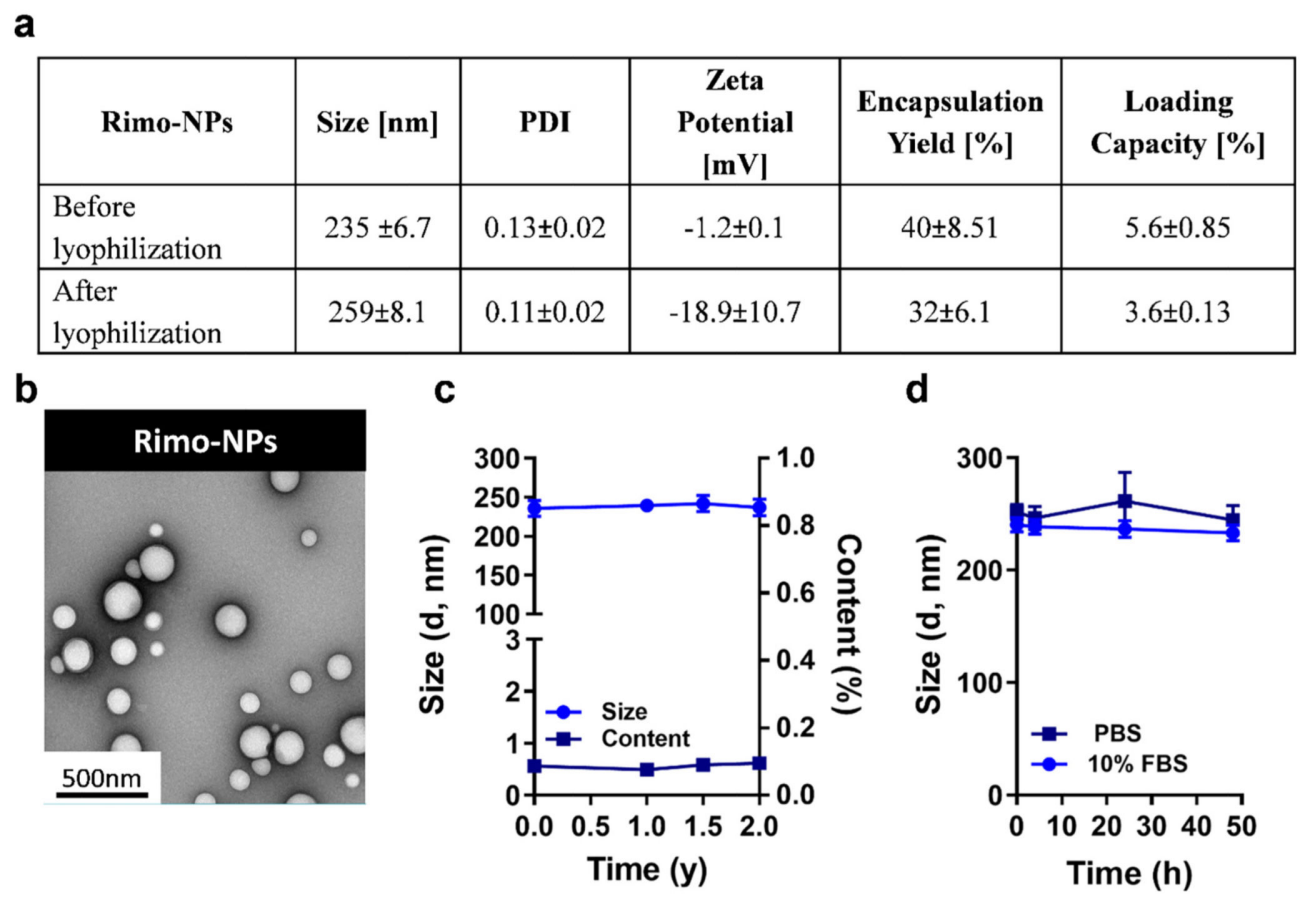
The physicochemical properties of Rimo-NPs. (a) Formulation. (b) Transmission electron microscopy (TEM) micrographs of Rimo-NPs immediately after lyophilization (Uranyl acetate negative staining, magnification 25 K, scale bar = 500 nm). (c) Long-term stability (size and content) of lyophilized Rimo-NPs stored at −20°C for 2 years. (d) Stability of Rimo-NPs in terms of size following incubation in 10% FBS solution in comparison to PBS solution. Data represent the mean ± SEM from 3 independent formulations per condition.

Ultimately, PLGA-based Rimo-NPs were successfully formulated and characterized with spherical geometry; they had a diameter size of ~250 nm, a relatively narrow PDI (~0.11 ± 0.02), a Z potential of −18.9 ± 10.7 mV, a loading capacity of 3.6 ± 0.13% rimonabant, and an encapsulation efficiency of 32.53 ± 6.1% after lyophilization (Fig. 1a-b). Before lyophilization, the NPs were washed with 10% albumin solution to remove loosely bound rimonabant (Supplementary Fig. 10). The physicochemical properties of Rimo-NPs, in terms of size and drug concentration, were not affected by lyophilization or changed after 2 years of storage at −20 °C (Fig. 1c, and Supplementary Fig. 10c). No significant protein adsorption on Rimo-NPs was detected following incubation in serum-containing media (10% FBS) in terms of size changes up to 48 h (Fig. 1d). The leakage of rimonabant from the NPs was low at 4 °C; it increased at 37 °C (Supplementary Fig. 10b). Rimonabant in the NPs was found to be in an amorphous state, indicated by differential scanning calorimetry (DSC); it exhibited a phase transition from the crystalline, rimonabant-HCl (Supplementary Fig. 11).

### 3.2 Increased peripheral accumulation of Rimo-NPs

**Fig. 2:**
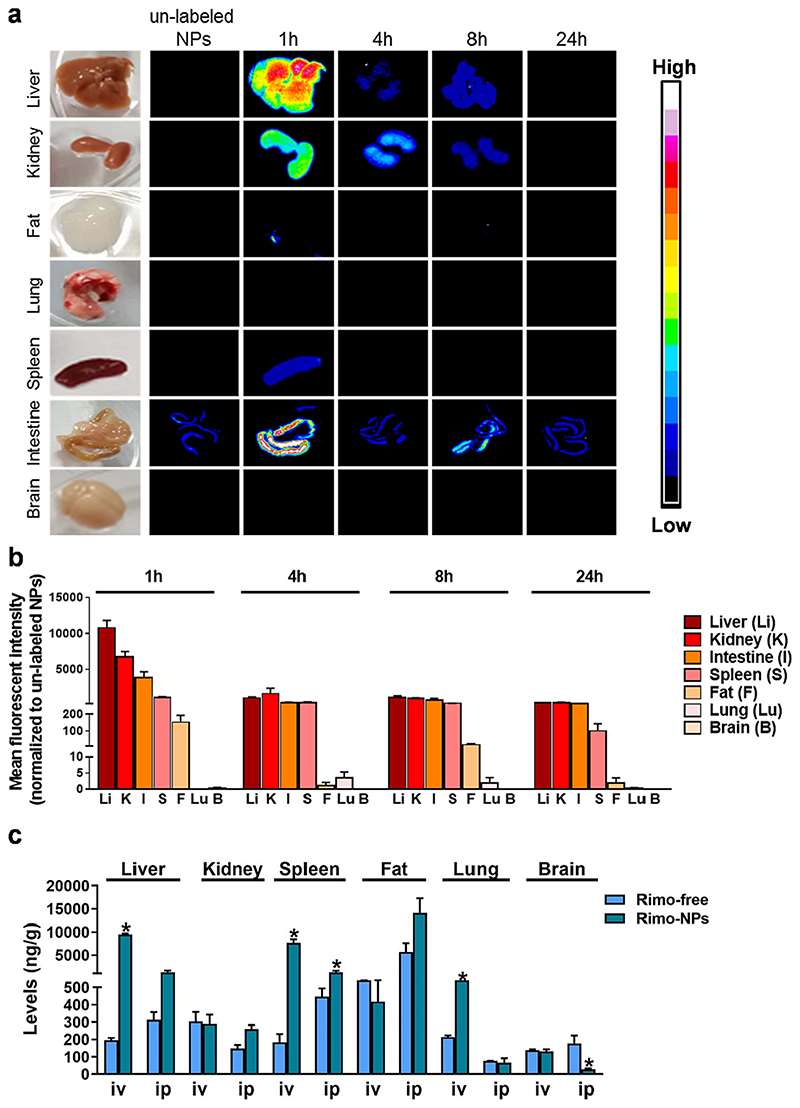
Rimo-NP biodistribution in mice. (a) Rimo-NPs (Cy5-PLGA labeled) were administered ip to C57Bl/6JHsd mice, and examined 1, 4, 8, and 24 h after injection. Representative organ micrographs harvested 1 h after treatment (left column) and scanning micrographs by means of a Typhoon scanner (right columns). (b) The quantified fluorescence intensities in each organ are normalized to unlabeled NP-treated animals. (c) The accumulation of rimonabant in organs was also evaluated by analyzing the rimonabant levels in the liver, kidney, spleen, fat, lung, and brain 1 h post-injection (1 mg/kg, ip or iv). Data represent the mean ± SEM of 3 mice per group. *p < 0.05 relative to free rimonabant levels in the same tissue and the route of administration.

To validate our hypothesis of rimonabant peripheralization by administering the drug embedded in NPs, we carried out thorough biodistribution studies. First, we determined the fate of Cy5-labeled NPs administered ipto mice. The NP biodistribution in the various organs was liver>kidney>intestine>spleen>fat>>> lungs; only a negligible amount was detected in the brain (Fig. 2a, b). In contrast, rimonabant formulated in the NPs with solutol or in liposomes (Supplementary Table 1) penetrated the brain. Noteworthy is the significantly higher accumulation of rimonabant in the liver and the spleen following treatment with Rimo-NPs, in comparison with animals treated with rimonabant solution (Fig. 2c; a 4-fold and 2.6-fold higher concentration in the liver and spleen, respectively). Remarkably, the brain levels of rimonabant were significantly (5-fold) lower following Rimo-NP administration, compared with free rimonabant solution, 1 h post-ip administration (Fig. 2c). In addition, high levels of rimonabant in the liver were found following iv or ip administration of Rimo-NPs (1 mg/ kg; Fig. 2c), as well as after various iv doses [0.01 to 3.0 mg/kg (Supplementary Fig. 12)]. The peripheralization of rimonabant, following Rimo-NP administration, was also manifested by the significantly higher levels of the drug in the lungs (1 mg/kg, iv) and in the spleen (0.01 to 1.0 mg/kg, iv), compared with free rimonabant treatments (Supplementary Fig. 12).

**Fig. 3:**
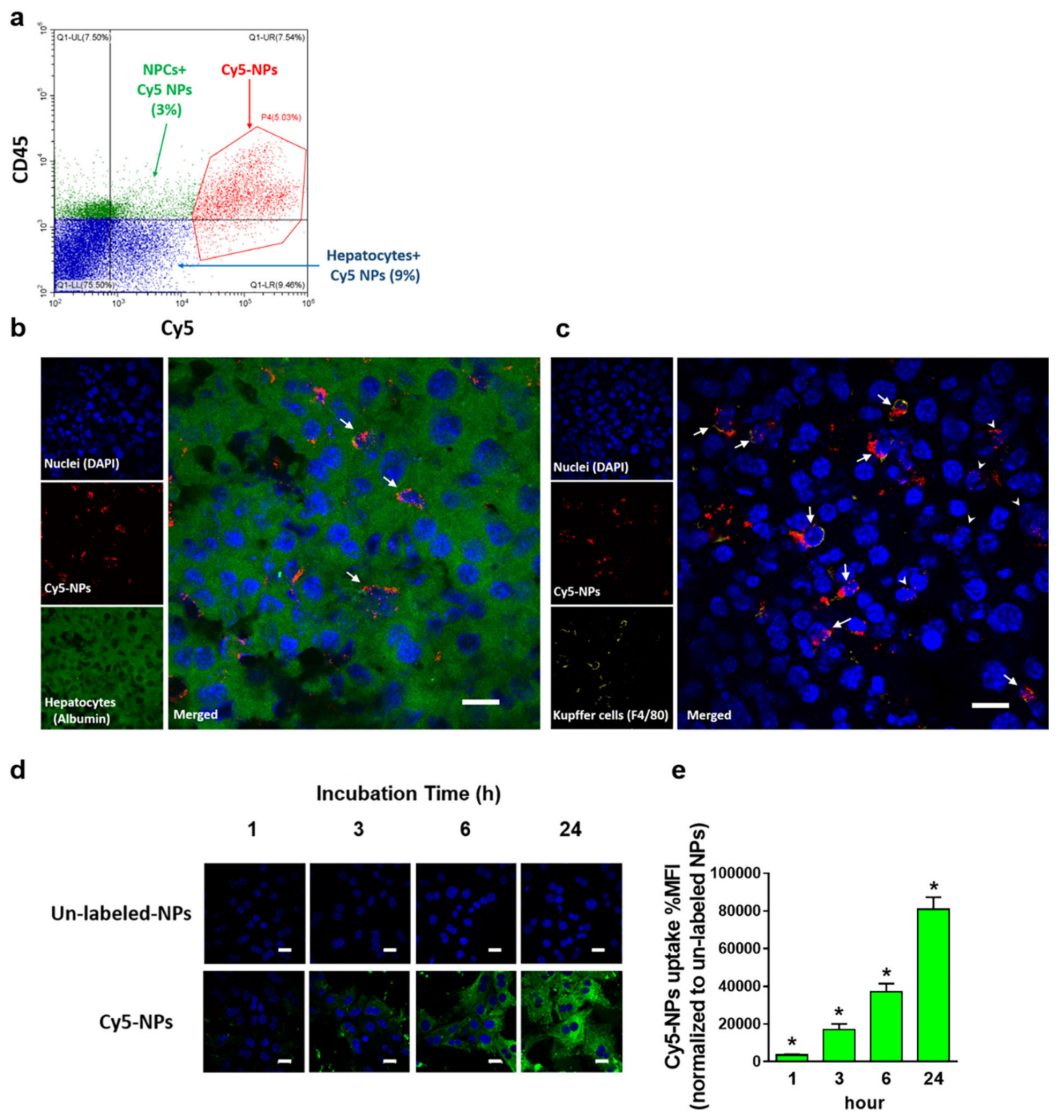
Rimo-NP uptake by liver cells. (a) FACS gating strategy demonstrating the increased accumulation of Rimo-NPs in the primary hepatocytes containing the parenchymal fraction (CD45-negative cells) as well as in NPCs (CD45-positive cells). (b) Representative images of Rimo-NP (Cy5-PLGA labeled) uptake by liver hepatocytes (arrows) in mice 1 h post-treatment (ip; 140 mg/kg NPs, 200 μL). (c) Representative image of Rimo-NP (Cy5-PLGA labeled) uptake by Kupffer cells (arrows) or non-Kupffer cells (arrowheads) in mice 1 h post-treatment (ip; 140 mg/kg NPs, 200 μL). For b and c, representative confocal microscopy images are shown [red, Rimo-NPs; green, hepatocytes (albumin-positive cells); dark yellow, Kupffer cells (F4/80-positive cells)], the co-localization of the NPs in hepatocytes or Kupffer cells is shown in the merged images. Magnification x60, scale bar = 20 μm. (d) Qualitative and (e) quantitative assessments of Rimo-NP (Cy5-PLGA labeled) uptake by primary mouse hepatocytes in culture [green, Cy5; blue, nuclei (DAPI)]. The fluorescence intensities are normalized to cells treated with unlabeled NPs; magnification x60, scale bar = 25 μm. Data represent the mean ± SEM of five independent experiments, *p < 0.05. (For interpretation of the references to colour in this figure legend, the reader is referred to the web version of this article.)

Since our targeted cells for therapy could be CB_1_R-expressing cells in the liver, e.g., hepatocytes or NPCs such as Kupffer cells, and due to the high levels of the drug found in the liver even 4 h after iv or ip administration of Rimo-NPs (Supplementary Fig. 13), we next assessed the specific cellular fate of Cy5-labeled NPs in the liver. As shown in Fig. 3a, an accumulation of Cy5-labeled NPs was found in 9% of primary hepatocytes containing a parenchymal fraction (CD45-negative cells), which were isolated from the animals 1 h after administration. The immunofluorescent analysis of liver sections, collected from mice injected with Cy5-labeled NPs, revealing that the NPs are indeed localized in hepatocytes (Fig. 3b), corroborated this finding. Additional support for Rimo-NPs’ affinity to hepatocytes is the increased uptake profile of Cy5-labeled NPs detected in primary mouse hepatocytes in vitro (Fig. 3d, e). Nevertheless, Rimo-NPs were also taken up by NPCs, since the NPs were found in a small fraction (3%) of CD45- or F4/80-positive cells, respectively (Fig. 3a, c). Of note is that the treatment was not associated with any hepatic cellular toxicity (Supplementary Fig. 14)

### 3.3 Rimo-NPs do not induce centrally mediated side effects

**Fig. 4:**
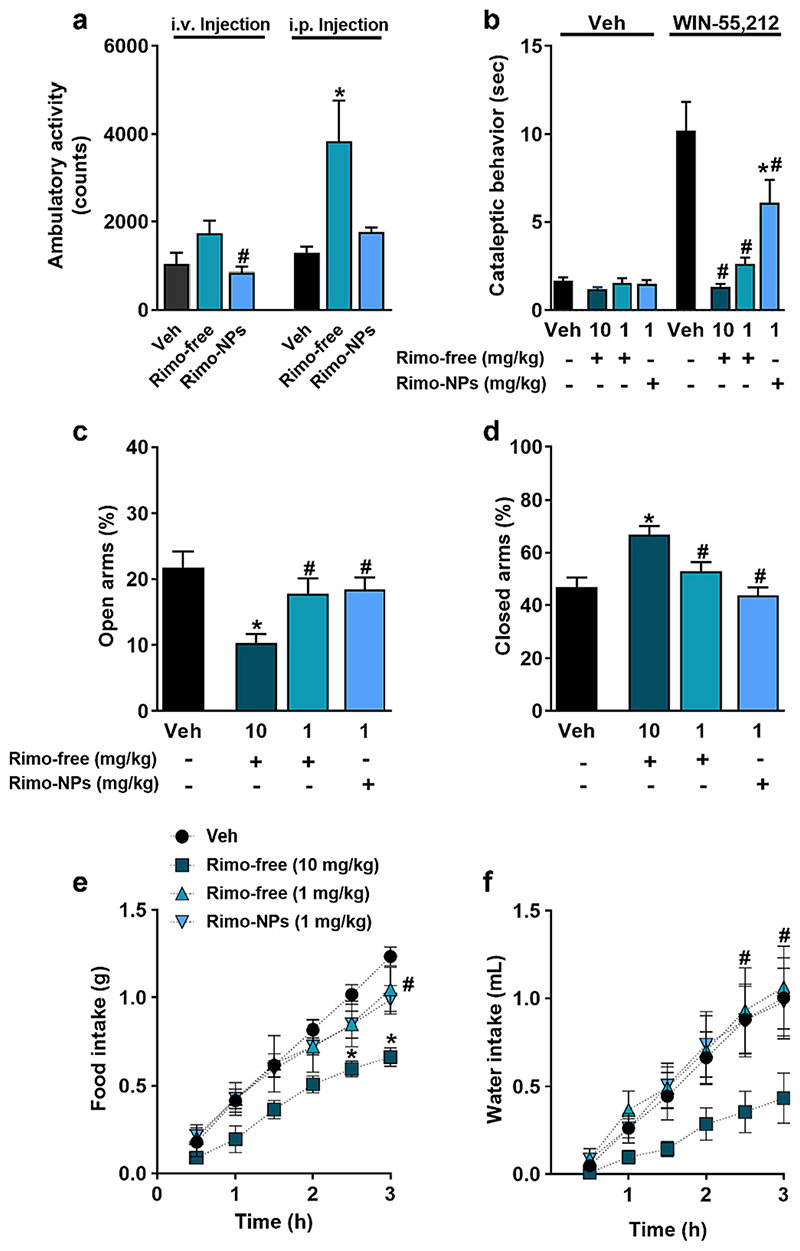
Rimo-NPs do not induce centrally mediated side effects. (a) Free rimonabant, but not Rimo-NPs increased the ambulatory activity both after ip and iv administration of 1 mg/kg. Data represent the mean ± SEM of 4 mice per group. *p < 0.05 relative to vehicle (Veh; free PLGA-NPs), ^#^*p* < 0.05 relative to free rimonabant. (b) Free rimonabant at 1 and 10 mg/kg ip, but not Rimo-NPs at 1 mg/kg ip, inhibited WIN-55,212 (3 mg/kg, ip)-induced catalepsy as measured by the bar assay. Data represent the mean ± SEM from 7 to 26 mice per group. *p < 0.05 relative to the corresponding vehicle (4% DMSO, 1% Tween80, 95% saline) w/o WIN-55,212, ^#^*p* < 0.05 relative to the corresponding vehicle with WIN-55,212. (c, d) Free rimonabant at 10 mg/kg ip, but not Rimo-NPs or free rimonabant, both at 1 mg/kg ip, induced an anxiogenic effect in the EPM. Data represent the mean ± SEM from 10 to 24 mice per group. *p < 0.05 relative to the corresponding vehicle (4% DMSO, 1% Tween80, 95% saline), ^#^*p* < 0.05 relative to free rimonabant at 10 mg/kg. (e, f) Free rimonabant at 10 mg/kg ip, but not Rimo-NPs or free rimonabant at 1 mg/kg ip, inhibited acute food and water intakes. Data represent the mean ± SEM from 4 mice per group. *p < 0.05 relative to the vehicle (Veh; free PLGA-NPs), ^#^*p* < 0.05 relative to free rimonabant at 10 mg/kg.

Encouraged by the distinctively different biodistribution of Rimo-NPs vs free rimonabant, we next determined whether the reduced brain levels of Rimo-NPs are associated with reduced behavioral side effects known to be mediated by blocking the brain’s CB_1_Rs. To that end, we used the 1 mg/kg dose for Rimo-NPs, since at this dose, high levels of rimonabant were found in the liver with negligible amounts found in the brain (Fig. 2). In those cases that free rimonabant at 1 mg/kg did not induce a robust central effect, we increased the dose to 10 mg/kg and used it as a “positive control” for CNS-mediated side effects. Whereas both ip and iv administrations of free rimonabant induced CNS-mediated hyperactivity, no such effect was found with Rimo-NPs (Fig. 4a). This effect was further validated in a dose-dependent study, demonstrating that only free rimonabant but not Rimo-NPs induced CNS-mediated hyperactivity administrated iv at doses of 0.1, 1, and 3 mg/kg or ip at doses of 1 and 3 mg/kg (Supplementary Fig. 15). We next assessed the ability of Rimo-NPs to antagonize the brain’s CB_1_R-induced catalepsy. Indeed, only free rimonabant (1 and 10 mg/kg, ip) blocked the cataleptic behavior mediated by WIN-55,212 (Fig. 4b). Interestingly, only the high dose of free rimonabant (10 mg/kg, ip) induced a robust anxiogenic response in the elevated plus maze (EPM) paradigm (Fig. 4c, d); it also inhibited acute food and water intakes in mice (Fig. 4e, f). All together, these findings suggest that the CB_1_R-mediated side effects of rimonabant are CNS-induced in a dose-dependent manner.

### 3.4 Inability of Rimo-NPs to affect the centrally mediated regulation of body weight and metabolic homeostasis

**Fig. 5:**
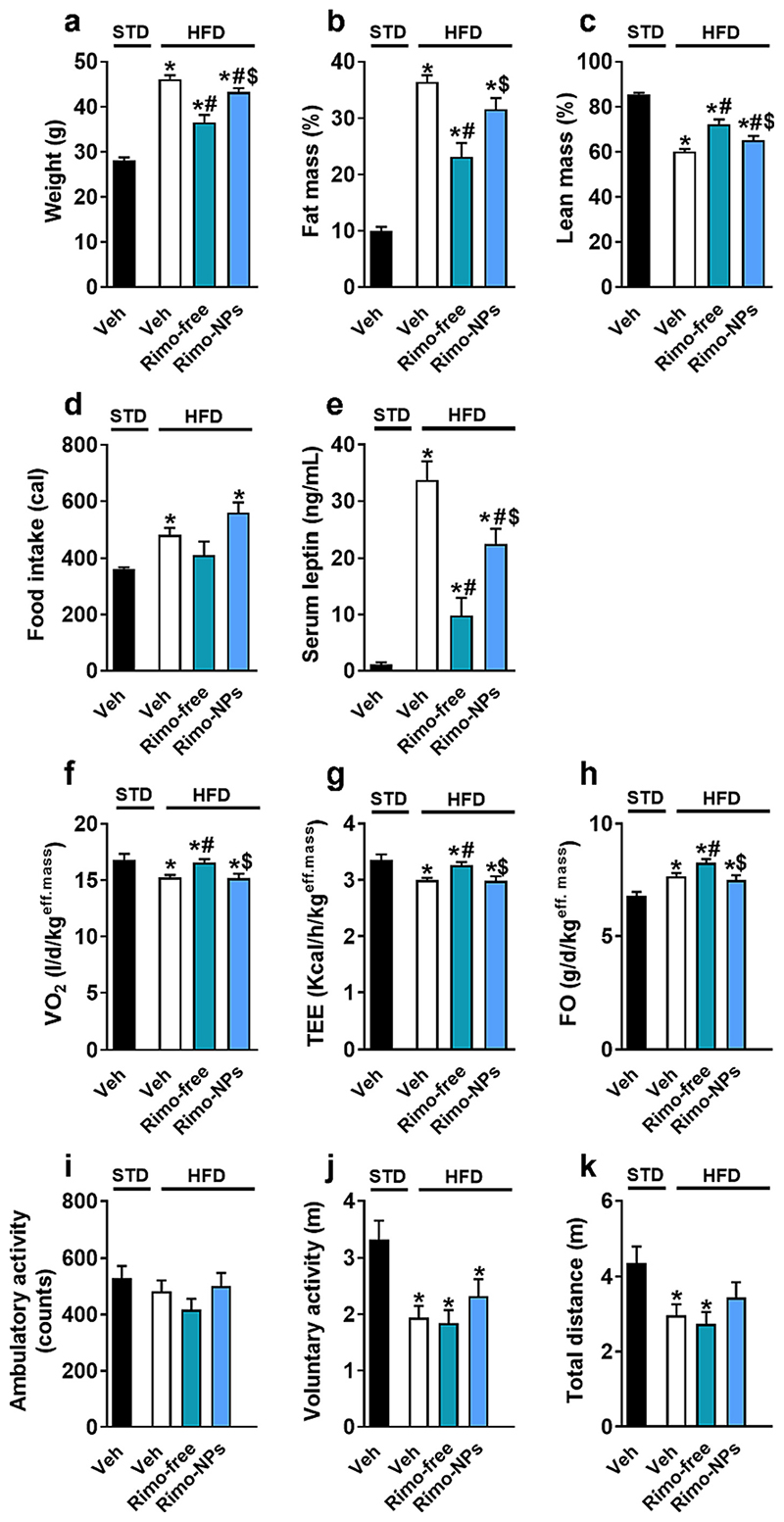
Metabolic effects of chronic treatment with free rimonabant or Rimo-NPs in DIO mice. Mice on STD or HFD for 14 weeks were treated with vehicle (Veh; free PLGA-NPs) or 1 mg/kg/d, ip, of free rimonabant or Rimo-NPs for 28 days. Free rimonabant significantly reduced body weight (a) and fat mass (b), increased lean mass (c), without affecting the food intake (d). Serum leptin levels were significantly reduced by free rimonabant and to a lesser extent by Rimo-NPs (e). Indirect calorimetry assessment over a 12 h period in the dark period revealed that free rimonabant but not Rimo-NPs resulted in an upregulation of oxygen consumption (VO_2_; f), total energy expenditure (TEE; g), and fat oxidation (FO; h) without affecting the ambulatory activity (i), voluntary activity (j), and the total distance the mice traveled in the cage (k). Data represent the mean ± SEM from 7 mice per group, *p < 0.05 relative to STD-Veh, ^#^*p* < 0.05 relative to HFD-Veh, ^$^*p* < 0.05 relative to HFD-free rimonabant.

The metabolic effects of Rimo-NPs and free rimonabant were next examined in a diet-induced obesity (DIO) mouse model (male C57BL/6 J) fed with a HFDfor 14 weeks. The obese mice were treated daily with Rimo-NPs in comparison to empty NPs (vehicle) and free rimonabant in solution (1 mg/kg/d, ip; for 28 days). Age- and sex-matched mice on a STDserved as controls. Following treatment with free rimonabant, and to a lesser extent with Rimo-NPs, the overweightness, increased adiposity, and reduced lean mass were significantly ameliorated (Fig. 5a-c). The minor effect exhibited by Rimo-NPs is most likely due to the poor penetration of rimonabant into the brain; it was unable to affect the CNS-induced reduction in food intake and to reverse hyperleptinemia (Fig. 5d, e). In addition, indirect calorimetry assessment at the end of the treatment regimen revealed that free rimonabant treatment, but not by Rimo-NPs, resulted in upregulation of oxygen consumption (VO_2_), total energy expenditure (TEE), and fat oxidation (FO) (Fig. 5f-h), without affecting the animal’s activity profile (Fig. 5i-k). These findings elucidate the notion that the modest improvement in body weight by free rimonabant is most likely due to the centrally-mediated increase in lipid oxidation.

### 3.5 Weight-independent effects of Rimo-NPs in ameliorating obesity-induced dyslipidemia, hepatic steatosis, and insulin resistance

**Fig. 6:**
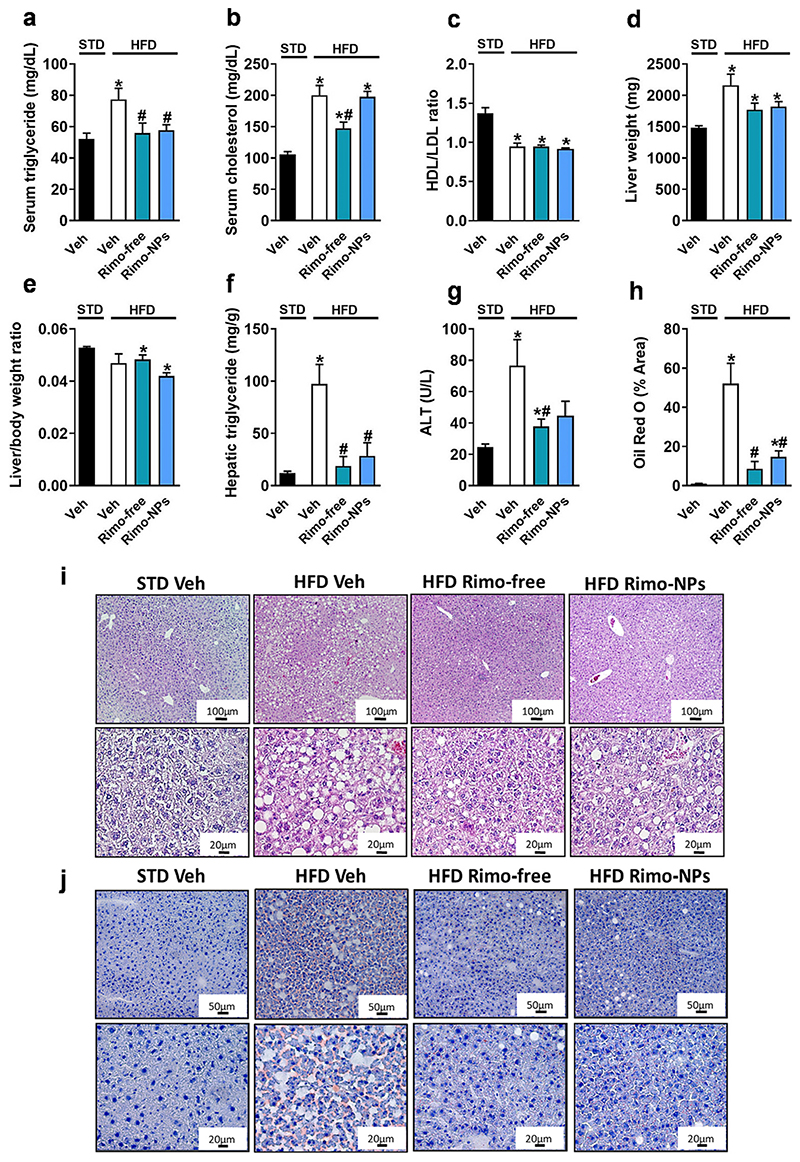
Weight-independent effects of Rimo-NPs in ameliorating obesity-induced dyslipidemia and hepatic steatosis. Mice on STD or HFD for 14 weeks were treated with vehicle (Veh; free PLGA-NPs) or 1 mg/kg/d, ip, of free rimonabant or Rimo-NPs for 28 days. (a) Serum triglycerides. (b) Serum total cholesterol. (c) The HDL-to-LDL ratio. Both free rimonabant and Rimo-NPs significantly reversed the HFD-induced hepatic steatosis, as measured by reductions in liver weight (d), liver-to-body weight ratio (e), hepatic triglyceride content (f), and circulating ALT levels (g). Fat deposition assessed by H&E (i) and Oil Red O (j) staining and quantification (h). Scale bar = 20 or 100 μm for H&E staining and 20 or 50 μm for Oil Red-O staining, as indicated on the images. Data represent the mean ± SEM from 7 mice per group, *p < 0.05 relative to STD-Veh, ^#^*p* < 0.05 relative to HFD-Veh, ^$^*p* < 0.05 relative to HFD-free rimonabant. (For interpretation of the references to colour in this figure legend, the reader is referred to the web version of this article.)

Next, we deciphered the effect of chronic treatment with Rimo-NPs, in comparison to free rimonabant, on obesity-induced dyslipidemia, hepatic steatosis, and insulin resistance. Our findings show that hyper-triglyceridemia, but not hypercholesterolemia, was ameliorated by Rimo-NP treatment (Fig. 6a, b). However, neither of the formulations affected the HDL/LDL cholesterol ratio (Fig. 6c). On the other hand, the HFD-induced fatty liver, reflected by increased liver weight, elevated hepatic triglyceride content, hepatocyte ballooning, fat accumulation, and hepatocellular damage (manifested by the elevated serum ALT levels), were completely reversed by either treatment (Fig. 6d-j). These positive effects are most likely attributed to the ability of rimonabant to increase hepatic fatty acid utilization/oxidation, as measured acutely in primary mouse hepatocytes exposed to lipotoxic conditions (0.5 mM O:P 2:1) and pre-treated with Rimo-NPs or free rimonabant (Supplementary Fig. 16), and chronically in DIO mice treated with 1 mg/kg for 28 days (Supplementary Fig. 16). Interestingly, in the acute condition both treatments significantly upregulated the expression levels of genes associated with fatty acid oxidation (*Cpta1*, *Acadm*, *Echs1*, *Hadh*, and *Acaa2*). However, only Rimo-NPs were found to enhance the expression of these genes in vivo, an effect that could be linked to the higher hepatic exposure of rimonabant found in these animals following chronic administration (Supplementary Fig. 17).

**Fig. 7:**
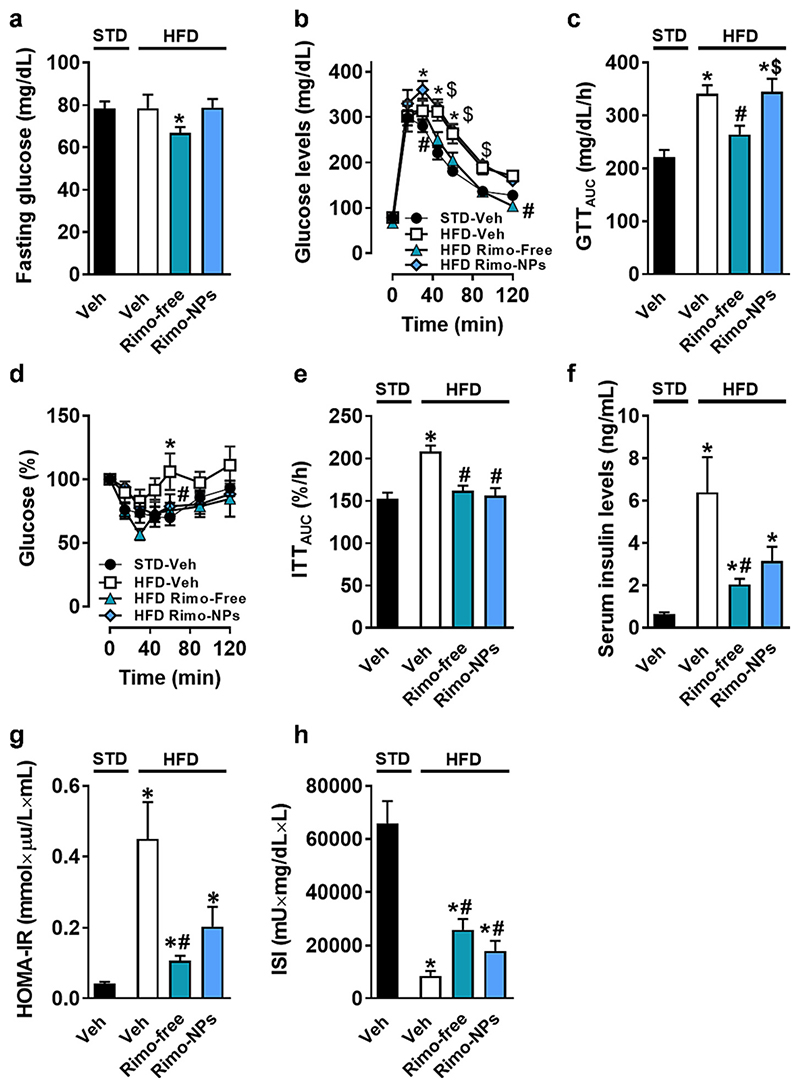
Weight-independent effects of Rimo-NPs in ameliorating obesity-induced insulin resistance. Mice on STD or HFD for 14 weeks were treated with vehicle (Veh; free PLGA-NPs) or 1 mg/kg/d, ip, of free rimonabant or Rimo-NPs for 28 days. Free rimonabant, but not Rimo-NPs, reversed obesity-induced glucose intolerance (a-c). Both Rimo-NPs and free rimonabant improved insulin sensitivity (d-e), reduced hyperinsulinemia (f), HOMA-IR (g), and ISI (h). Data represent the mean ± SEM from 7 mice per group, *p < 0.05 relative to STD-Veh, ^#^*p* < 0.05 relative to HFD-Veh, ^$^*p* < 0.05 relative to HFD-free rimonabant.

Free rimonabant treatment resulted in both improved obesity-induced glucose intolerance (Fig. 7a-c), and improved insulin sensitivity (Fig. 7d-h). In contrast, treatment with Rimo-NPs affected only insulin sensitivity, reflected by the reduced glucose levels, following a bolus of insulin, reduced hyperinsulinemia, and improved HOMA-IR and ISI levels (Fig. 7d-h). In addition, the extensive accumulation of Rimo-NPs in the kidney (Fig. 2) could explain the greater effect of normalizing the water consumption-to-urine excretion ratio (Supplementary Fig. 18). Congruently with the metabolic efficacy of Rimo-NPs, the levels of rimonabant measured 18 h following the last injection of the novel formulation, in comparison to free rimonabant treatment, to HFD-fed mice were significantly higher in liver, kidney, spleen, and blood (with a similar trend found in fat and lungs, which did not reach statistical significance). Comparable low levels of rimonabant were found in the brain in the two treatment groups (Supplementary Fig. 17). These findings further support the peripheral diversion of rimonabant by the NPs, resulting in improved metabolic abnormalities associated with obesity.

## 4 Discussion

Mounting evidence supports CB1R antagonism as a key pharmacological mechanism to block the overactivation of the eCB signaling system for the treatment of obesity and its cardiometabolic complications, such as NAFLD and T2D. Consequently, the observation that the eCB/CB1R system is overactive in humans with obesity and in genetic- and diet-induced obese animals [7,11–16,30,38–41] led to preclinical developments and clinical efforts to test CB_1_R antagonists for the treatment of the metabolic syndrome. Importantly, the first-in-class synthetic CB_1_R inverse agonist, rimonabant, was found to reduce weight gain and food intake in a dose-dependent manner under both fasting and non-fasting conditions [42–46] as well as to inhibit the motivation for palatable food [47,48]. These data, together with the fact that CB_1_R knockout mice are hypophagic and lean [8], promoted the clinical testing of rimonabant in humans. Indeed, when it was tested in obese/overweight individuals with the metabolic syndrome, rimonabant was found to effectively reduce food intake and body weight, reverse obesity-induced insulin and leptin resistance, decrease hepatic steatosis and liver injury, and improve glucose homeostasis and hyper-lipidemias [18–20,49–52]. These findings led to its clinical approval by the European Medicines Agency (EMA) in 2006, under the name of Acomplia® (Sanofi-Aventis), for the treatment of obesity and its related metabolic risk factors in non-diabetic and diabetic overweight and obese patients. However, growing evidence of anxiety, depression, and suicidal ideation, reported in a small but significant portion of individuals treated with rimonabant [53], led to its eventual withdrawal from the market in 2009. Prompted by the risk of these CNS-mediated adverse effects, several pharmaceutical companies that had been developing proprietary CB1R blockers terminated their ongoing clinical trials. Moreover, concerns have been raised regarding the therapeutic potential of this class of molecules in modulating the eCB/CB_1_R signaling system for the treatment of obesity and its metabolic abnormalities [54].

The lack of effective medications for treating the metabolic syndrome in general, on one hand, and the key physiological and pathological roles that the eCB/CB_1_R system plays, on the other hand, as well as evidence on the specific deletions of CB_1_R in the liver, adipose tissue, kidney, pancreas, and skeletal muscle, have underscored its importance in modulating peripheral metabolic function [28,29,39,40,55–57]. Therefore, several novel strategies have been suggested and explored to mitigate/eliminate the CB_1_R-induced CNS psychiatric side effects, while retaining the therapeutic benefit of CB_1_R blockade. These approaches include developing CB_1_R neutral antagonists (e.g., PIMSR, AM4113, and NESS06SM), peripherally restricted CB_1_R antagonists (e.g., AM6545, JD5037, MRI-1867, and TM-38837), CB_1_R allosteric modulators (e.g., Org27569, PSCNABM-1, and Pepcans), as well as monoclonal CB_1_R antibodies (Nimacimab and IM-10) [for further reading, see [58–60]]. Here, we describe an alternative strategy in which a novel drug delivery system was used as a tool for the peripheralization of rimonabant for the treatment of NAFLD and T2D. We hypothesized that encapsulating rimonabant in NPs for its hepatic distribution would allow us to create the “next generation” of drugs that would target the CB_1_R receptor only in the liver, without the side effects associated with blocking the same receptor in the brain. Indeed, by using a nano-drug delivery system, we were able to successfully divert the centrally acting and water-insoluble CB1R blocker, rimonabant, to the liver, reducing its toxic centrally mediated side effects. In this study, we further demonstrated its therapeutic potential to reduce obesity-induced hepatic steatosis and liver injury, improve insulin sensitivity, and reverse hypertriglyceridemia.

As a proof-of-concept for a nano-scaled drug delivery system, we experimented with various formulations (Supplementary Text, Supplementary Figs. 1–9, and Supplementary Tables 1–8). Unfortunately, in our preliminary trials, including use of various polymers, lipids, and surface-active agents, as well as several different methods of NP formulation, we were unable to efficiently entrap rimonabant in nano-drug delivery systems and/or to prevent it from penetrating into the brain. These findings are in accordance with a previous study by Esposito and colleagues, who described the encapsulation of rimonabant in a nanostructured lipid carrier for intranasal delivery in order to bypass the BBB and to target brain CB_1_Rs [61]. Here, we took a unique approach to limit the brain penetration of rimonabant and retain its peripheral metabolic actions. The single emulsion evaporation approach used here resulted in a stable formulation with high loading capacity and encapsulation efficiency of rimonabant in the NPs. The loosely bound molecules on the surface of the NPs were successfully removed by washing the formulation with an albumin solution, since rimonabant efficiently binds to proteins in the blood [62]. The optimal Rimo-NPs exhibited a retarded release of rimonabant, allowing the drug to remain inside the NPs while circulating in the blood, before reaching peripheral tissues. The ultimate fate of all non-targeted nanomedicines is similar, demonstrating enhanced NP uptake by the liver and spleen [63–67]. In addition, our data suggest that drug release from the NPs takes place in these organs rather than in the circulation during the first hour after administration. This is well correlated with the biodistribution analyses as well as the negligible side effect profile recorded during the first hour following the administration of Rimo-NPs.

Peripheral targeting of CB_1_R antagonists without compromising CNS safety is mandatory when considering this approach for the therapeutic benefits. Ideally, the actual drug levels in the brain should be as low as possible, and the ratio of a target organ exposure to brain exposure for improving CNS safety is another important pharmacokinetic parameter to be considered [60]. Our novel nano-drug formulation complies with both of these features, displaying significantly low levels of rimonabant in the brains of mice acutely injected with Rimo-NPs in comparison with free rimonabant, as well as high liver-to-brain ratios (~50 to 100, measured by LC-MS/MS or fluorescent-labeled NPs). Comparing the levels of rimonabant in the target organs, which revealed dose- and time-dependent accumulation in mouse liver and hepatocytes treated with Rimo-NPs, allowed us to reduce the therapeutic dose of rimonabant to 1 mg/kg, which also contributed to the negligible levels of the drug found in the brain. A similar tissue biodistribution profile was also found upon chronic administration to HFD-fed mice treated for 28 days with the nano formulation. At that dose (administered iv or ip), Rimo-NPs did not cause CNS-mediated side effects. By using the EPM paradigm, we showed that only a high dose of free rimonabant (10 mg/kg), but not free rimonabant or Rimo-NPs at 1 mg/kg, induces a robust anxiogenic response, manifested by the enhanced time that the mice spent in the closed arms and the reduced time that they spent in the open arms of the maze. These findings, together with the inability of Rimo-NPs at 1 mg/kg to induce centrally mediated hyperambulation, to reverse CB_1_R-induced cataleptic behavior, and to inhibit fasting-induced food and water intakes, clearly suggest that while entrapped in NPs, rimonabant is unlikely to induce centrally mediated side effects in mice. Indeed, it has been shown that a low-concentration pharmacological treatment with rimonabant coincides with potent antagonism of CB_1_R-mediated G protein activation in vivo, whereas high doses of rimonabant are consistent with its inverse agonism profile, which contributes to the existence of brain-induced adverse effects [68]. Interestingly, although rimonabant is known to induce depression in humans, in the experimental trials used thus far to evaluate its ability to mediate depressive-like symptoms have concluded that this side effect cannot be accurately measured in mice. Specifically, Marinho et al. showed that free rimonabant at 10 mg/kg did not induce depressive-like symptoms in the Forced Swim Test [69]. Moreover, Gamble-George and colleagues showed that free rimonabant at 3 and 10 mg/kg did not induce despair-like behavior in the Tail Suspension Test [70]. Since Rimo-NPs were given at a dose of 1 mg/kg, the risk of depression-like behaviors induced by the nano formulation are relatively low.

An apparently unexpected observation was the accumulation of the Rimo-NPs in the kidney. In fact, reports on NPs localizing in the kidneys are rare in the literature. The majority of untargeted NPs (<200 nm) primarily tend to accumulate in the visceral organs, liver and spleen, either via circulating mononuclear phagocyte system trafficking or via liver fenestrations (approximately 100 nm) [63–67]. Microparticles (with diameters above 1000 nm) often localize in the lungs due to entrapment in pulmonary capillary beds. In a recent study by Williams et al., [71] it was found that the low opsonization potential of the NPs is a critical parameter responsible for kidney accumulation. Therefore, it can be suggested that specific opsonization of the NPs affected their uptake in the kidneys also here. In addition, their decay over a course of 24 h may suggest that the NPs are eliminated via the kidneys. It should be noted that rimonabant was also accumulated in the kidney (~300–400 ng/g) in parallel with the presence of NPs in the kidney. However, additional work is needed to further clarify this observation.

Underscoring the contribution of central vs. peripheral CB_1_R to the development of obesity and its reversal by CB_1_R blockers is a key issue in the eCB field. By using a very low dose of rimonabant encapsulated in NPs that do not cross the BBB, we clearly distinguish between weight loss-dependent and -independent mechanisms regulated by CB1R blockade. Our data indicate that blockade of central CB_1_Rs is essential for the anti-obesity and improved metabolic efficiency of rimonabant, which was found effective in reducing body weight, fat mass, hyper-leptinemia, and in increasing the total energy expenditure and fat oxidation. These effects, reported previously with higher doses of rimonabant (3 and 10 mg/kg) [32,44,72–74], are most likely mediated via blockade of CB_1_R in the CNS; this was reflected in our study by a lower dose of the free drug (1 mg/kg), manifested in high brain exposure. Interestingly, the reduced metabolic effects of Rimo-NPs on body weight, fat mass, and energy utilization are most likely mediated via the combined result of rimonabant’s low brain exposure and a reduction in its systemic presence in tissues, such as adipose tissue and the GI tract, which are known to contribute to weight management. On the other hand, our data indicate that blockade of peripheral CB_1_R (mainly in the liver), independent of weight loss, is sufficient to ameliorate obesity-induced hepatic steatosis and liver injury, insulin resistance, and hypertriglyceridemia; these effects are known to be mediated via blocking the CB_1_R in hepatocytes [10,26,27,32,39,75–79]. These findings support the critical role of hepatic CB_1_R in regulating these metabolic parameters, and further suggest that targeting hepatic CB_1_R can be considered a valid therapeutic approach for treating NAFLD and T2D.

In light of the above-mentioned role of hepatocyte CB_1_R, a recent study by Wang and colleagues raised concerns about the reproducibility of the effects seen in hepatocyte-specific CB_1_R^-/-^ mice, by demonstrating that deletion of CB_1_R in hepatocytes did not alter de novo lipogenesis, insulin resistance, or the development of NAFLD in response to an HFD [80]. With knowledge of this discrepancy, one should also consider that besides hepatocytes, CB_1_Rs have also been reported in NPCs such as stellate cells [81,82], Kupffer cells [83,84], hepatic myofibroblasts [83], and hepatic vascular endothelial cells [85–88]. Their contribution to hepatic steatosis, insulin resistance, NASH, hepatic fibrosis and cirrhosis is well documented (reviewed in [83,89–91]). In line with these findings, submicron-sized NPs (100–1000 nm) are known to be sequestered by both hepatocytes and NPCs, such as Kupffer cells [63–67] [92,93]. To elucidate the intrahepatic distribution of Rimo-NPs, we used both FACS analysis on isolated mouse hepatocytes and NPCs, in addition to immunofluorescence. Whereas our data indicate that Rimo-NPs are largely taken up by hepatocytes, a significant, portion of NPs were also taken up by NPCs. These findings may further contribute to the improved metabolic phenotype of mice treated with the nano formulation, supporting our approach. Moreover, Rimo-NPs taken up by Kupffer cells could release the drug blocking CB_1_R in adjacent hepatocytes. An additional mechanism could be that even if Rimo-NPs are delivered intracellularly to hepatocytes or NPCs, once released from the NPs, rimonabant could block the receptor in the mitochondria rather than in the cell membrane. It is worth mentioning that the metabolic effects of rimonabant (as the ‘first-in-class’ CB_1_R blocker) on hepatic lipid metabolism and insulin resistance have been vastly studies and described earlier by multiple groups, including us [76,77,83,94–105]. Our current data showing that chronic treatment of obese mice with Rimo-NPs increased the expression levels of fatty-acid β-oxidation genes further support the well-known positive effect of CB_1_R blockade on hepatic lipid metabolism. Nevertheless, further work is required to delineate the role of a specific cell type within the liver or by CB_1_R located intracellularly within these cells, and to determine the effectiveness of CB1R blockade in improving hepatic steatosis and the insulin resistance.

## 5 Conclusions

The danger posed by NAFLD, as a sign of more serious conditions such as insulin resistance and T2D, is increasingly recognized; however, there is a dearth of pharmacological agents that are effective in the treatment of NAFLD and T2D. Overactivation of the eCB/CB_1_R system is thought to play a significant role in the development of obesity and its metabolic abnormalities, such as NAFLD and T2D. Therefore, blockade of CB1R represents a promising approach to potentially reverse these pathologies. However, the clinical use of globally acting CB_1_R blockers (e.g., rimonabant, taranabant, ibipinabant, and otenabant) was halted largely due to their centrally mediated side effects. Whereas several chemical approaches to mitigate brain penetration of these drugs have been recently raised and preclinically validated, a strategy in which a nano-drug delivery system could be utilized to retain the drug’s metabolic efficacy without jeopardizing CNS safety has never been reported. Our data show encouraging results for the encapsulation of small amounts of the drug in NPs for therapeutic benefits. When administered to mice in a dose that neither crosses the BBB nor causes side effects, high levels of rimonabant were found in the liver. In addition, the liver-targeted version of rimonabant could ameliorate diet-induced hepatic steatosis as well as insulin resistance in obese mice. This work and its novel findings have important translational/therapeutic implications. Nevertheless, future studies of this unique delivery system should focus on designing an appropriate formulation of these NPs for oral, subcutaneous, or intramuscular delivery to facilitate its practical clinical translation and application.

## Supplementary Material

Supplementary Data

## Data Availability

Data will be made available on request.
