# Supplementary material for: Hepatic targeting of the centrally active cannabinoid 1 receptor (CB1R) blocker rimonabant via PLGA nanoparticles for treating fatty liver disease and diabetes
Source: J Control Release. Author manuscript; Available in PMC 2023 Sep 7. (PMC9900386; doi:10.1016/j.jconrel.2022.11.040)
Supplement: Supplementary Data [file EMS186828-supplement-Supplementary_Data.pdf]

Supporting information

Hepatic Targeting of the Centrally Active Cannabinoid 1 Receptor (CB1R) Blocker Rimonabant via PLGA Nanoparticles for Treating Fatty Liver Disease and Diabetes

Shira Hirsch[1], Liad Hinden[1], Meital Naim[2], Saja Baraghithy[1], Anna Permyakova[1], Shahar Azar[1], Taher Nasser[2], Emma Portnoy[3], Majd Agbaria[2], Alina Nemirovski[2], Gershon Golomb[2], and Joseph Tam[1,*]

[1]Obesity and Metabolism Laboratory, [2]The Institute for Drug Research, School of Pharmacy, Faculty of Medicine, The Hebrew University of Jerusalem, Israel; [3]Department of Biochemistry, Institute for Medical Research Israel-Canada, Hebrew University-Hadassah Medical School, Israel.

Supplementary Table 1. Summary of formulation attempts to encapsulate rimonabant
Supplementary Figure 1. Cryo-TEM images of the initial formulation made by nanoprecipitation
Supplementary Table 2. Formulations prepared by nanoprecipitation for *in vivo* administration
Supplementary Figure 2. Brain and circulating levels of rimonabant (nanoprecipitation)
Supplementary Figure 3. Release of rimonabant base from PLGA 16kDa acid-terminated NPs, prepared using a single- or double-emulsion evaporation technique
Supplementary Table 3. Amounts of rimonabant-base (μg) encapsulated in NPs (PLGA 16 kDa, prepared by an emulsion evaporation technique) before and after washing the NPs with a 10% albumin solution
Supplementary Figure 4. Release of rimonabant-base from 100 kDa ester-terminated PLGA NPs prepared using a single- or double-emulsion evaporation technique
Supplementary Table 4. Amounts of rimonabant-base (μg) measured in NPs before and after washing the NPs with a 10% albumin solution.
Supplementary Figure 5. Release of rimonabant-HCl from 100 kDa ester-terminated PLGA NPs prepared using a single- or double-emulsion evaporation technique

Supplementary Table 5. Amount of rimonabant-HCl (μg) encapsulated in NPs (ester-terminated PLGA 100 kDa, prepared by an emulsion evaporation technique) before and after washing the NPs with a 10% albumin solution

Supplementary Figure 6. Release test of rimonabant-base from 50 kDa acid-terminated PLGA NPs prepared using the single- or double-emulsion evaporation technique

Supplementary Table 6. Amounts of rimonabant-base (μg) encapsulated in NPs (acid-terminated PLGA 50 kDa, prepared by an emulsion evaporation technique) before and after washing the NPs with a 10% albumin solution.

Supplementary Figure 7. Release of rimonabant-HCl from 50 kDa acid-terminated PLGA NPs prepared using single- or double- emulsion evaporation techniques

Supplementary Table 7. Amounts of rimonabant-HCl (μg) encapsulated in NPs (acid-terminated PLGA 50 kDa, prepared by an emulsion evaporation technique) before and after washing the NPs with a 10% albumin solution.

Supplementary Table 8. Liposomal formulations.

Supplementary Figure 8. Cryo-TEM images of the liposomes.

Supplementary Figure 9. Brain and circulating levels of rimonabant (liposomes)

Supplementary Figure 10. Stability assessment of Rimo-NPs

Supplementary Figure 11. DSC thermogram of rimonabant base, rimonabant HCl, Blank-NPs, and Rimo-NPs

Supplementary Figure 12. Tissue levels of Rimonabant after iv administration

Supplementary Figure 13. Rimonabant levels in the liver 4- and 24-hours following iv or ip administration.

Supplementary Figure 14. Toxicity of Rimo-NPs

Supplementary Figure 15. Inability of Rimo-NPs to induce CNS-mediated hyperactivity

Supplementary Figure 16. Rimo-NPs enhances hepatic fatty acid utilization/oxidation

Supplementary Figure 17. Rimo-NP biodistribution in diet-induced obese mice

Supplementary Figure 18. Water consumption-to-urine secretion ratio following chronic treatment with Rimo-NPs

Supplementary Table 9. Mouse Primers used for RT-PCR Analysis

Supplementary Table 1. Formulation attempts to encapsulate rimonabant.

Type of NPs	Method	Rimonabant Base/Hcl	Ingredients	Surfactant	Encapsulation efficacy (%)	Loading capacity (%)	Size and PDI	Brain penetration
Polymeric nanoparticles	Nanoprecipitation	Base	PLGA 50:50 70kDa, ester terminated	Solutol HS 15	44	6.7	111.4±0.15	+++
		Base	PLA 23kDa, ester terminated	Solutol HS 15	50	7.6	117.8±0.11	+++
		Base	PLGA 50:50 100kDa, ester terminated	Solutol HS 15	58	8.7	121.3±0.14	+++
		Base	PLGA 75:25 100kDa, ester terminated	Solutol HS 15	45	6.8	135.6±0.11	+++
	Single emulsion evaporation (data after albumin wash)	Base	PLGA 50:50 100kDa, ester terminated	2% PVA	10	1.0	229.3±0.10	N/A
		HCl	PLGA 50:50 100kDa, ester terminated	2% PVA	32	3.2	250±0.15	N/A
		Base	PLGA 50:50 50kDa, acid terminated	2% PVA	63	6.3	245.9±0.10	N/A
		HCl	PLGA 50:50 50kDa, acid terminated	2% PVA	56	5.6	232±0.13	+
	Double emulsion evaporation (data after albumin wash)	Base	PLGA 50:50 100kDa, ester terminated	2% PVA	6	0.6	N/A	N/A
		HCl	PLGA 50:50 100kDa, ester terminated	2% PVA	23	2.3	254.6±0.15	N/A
		Base	PLGA 50:50 50kDa, acid terminated	2% PVA	40	4.0	269±0.18	N/A
		HCl	PLGA 50:50 50kDa, acid terminated	2% PVA	47	4.7	N/A	N/A
Liposomes	Liposomes preparation from microemulsion	HCl	POPC, cholesterol, Phosphatidylglycerol, DSPE-PEG	N/A	89-93	1.2	130±0.11	+++

PLGA, Poly Lactic-co-Glycolic Acid. Poly(DL-lactide-*co*-glycolide) 50:50, 75:25-ratio between glycolic to lactic acid in the polymer chains. PLA, Poly Lactic Acid. POPC, 1-palmitoyl-2-oleoyl-sn-glycero-3-phosphocholine. DSPE-PEG, 1, 2-Distearoyl-sn-glycero-3-phosphoethanolamine-Poly(ethylene glycol), PDI, poly dispersity index.

Summary of formulation attempts to encapsulate Rimonabant

Rationale

The purpose of the best final formulation was to encapsulate the drug of choice in NPs at a high loading capacity, and with a high encapsulation efficacy, so that the formulation will exhibit high stability. In addition, the NPs should be greater than 100 nm in size, and bear a negative or neutral zeta potential, and the suspension should have a small amount of free drug in it. Lastly, the formulation needs to encapsulate the drug in a way that it will not be released into the circulation in the first hour after injection, to prevent brain penetration before the NPs distribute into peripheral organs.

To craft a proper drug delivery system, we used a variety of polymers using different techniques [1].

Nanoprecipitation

NPs were prepared using the interfacial deposition method [2]. PLGA (complementary to 100 mg of various MWs), oleyl cysteine amide (OCA) cross-linker (0, 5, 10, 15, and 20 mg), rimonabant base (15 mg), and MCT oil (0, 7.5, 15, and 30 mg) were dissolved in acetone (25 mL). Solutol HS 15 (10-50 mg) was dissolved in water (50 mL). The organic phase was added dropwise to the aqueous phase with stirring using a Hei-TORQUE 200 stirrer (Heidolph). The formulation's volume was reduced to 10 mL by evaporation (25°C) at reduced pressure on a Rotavapor (Laborota 4000 efficient, Heidolph Instruments).

Briefly, the PLGA polymer was dissolved in different amounts of acetone ranging between 35 mg and 99 mg with the complement of the other ingredients (up to 100 mg): rimonabant, OCA, and MCT oil. The organic phase was poured into an aqueous phase, which contained 0.1%-0.02%

w/v Solutol® HS 15. The volume ratio between the organic and aqueous phases was 1:2 v/v. The suspension was stirred at 900 rpm for 20 min, and then acetone was removed by reduced pressure evaporation. For a concentrated formulation, water was also vaporized until the desired final volume (10 mL) was achieved. The NPs were purified by centrifugation (4000 rpm, for 5 min, at 25°C).

An example of a formulation prepared by the nanoprecipitation method: The acid-terminated 15 kDa PLGA (50:50 LA:GA) was examined for its loading capacity of the drug in the presence of different amounts of OCA. The formulations exhibited a low average diameter (<100 nm) with a low size distribution (PDI<0.1), likely rendering them unsuitable for iv injection because of renal clearance. The *zeta* value was low (-20 mV), indicating the presence of aggregates that may have formed over time. The maximal amount of rimonabant in the suspension was ~5%, and it was lower (3.3%) in the nanosphere. In parallel, a huge precipitate formed immediately. The formulations were characterized by low drug stability, i.e., 1-2 days following their preparation, precipitate was already present, indicating their instability and the need for immediate lyophilization. In addition, the percentage of free drug was around 20%.

Optimization of the formulations prepared by nanoprecipitation: To optimize the loading capacity, encapsulation efficacy, and high stability, a few dozen formulations were prepared. Each time, a different parameter was amended, e.g., the type of PLGAs, different molecular weights of PLGAs, the ratio between glycolic and lactic acid (LA:GA), acid-terminated vs. ester-terminated polymer, testing the surfactant effects (OCA; Solutol), and MCT oils.

A summary of the results is presented below:

1. *Adding OCA - Increasing the loading capacity of rimonabant in the formulation and the stability of formulation.* By adding OCA, an amphiphilic linker molecule, a derivative of oleic acid, is functionalized with a polar thiol group contributed by cysteine. The dual character of this molecule enables anchoring inside the polymeric matrix core via the lipophilic oleyl chain, while the polar group is docked on the surface of NPs, facilitating thiol functionalization of the surface and the subsequent conjugation to any succinimidyl maleimide-activated molecular moiety [3]. The formulations exhibited a larger average diameter (>120 nm) with a low size distribution (PDI<0.15), and a zeta value of -40 mV, indicating stability over time. The formulation was able to load ~10% rimonabant and remained stable for ~10 days. However, examination of the supernatant after sedimentation of the NPs showed that the amount of rimonabant in the nanosphere was ~6% and that the rest (almost 40% of the amount in the suspension) was freely floating in the solution. There was no second sedimentation of rimonabant in the formulation even 10 days following NP preparation.

2. *Types of PLGAs - Increasing the loading capacity and stability.* Similarly, increasing the weight of PLGA to 100 kDa, and changing its end to an ester termination resulted in a dramatic increase in the loading capacity of rimonabant to ~8%. The ester-terminated PLGA, with a higher proportion of lactic acid and a higher MW, increases the hydrophobicity features; this has the potential to more effectively encapsulate the hydrophobic compounds [4, 5]. In a formulation containing an ester-terminated 70 kDa PLGA together with a 23 kDa PLA, there was even more improvement, to ~10% LC of rimonabant, and a second sediment formed 10 days after the preparation was created.

3. *Adding MCT oil - Increasing stability.* MCT oil was added to the formulation to create a hydrophobic core that dissolves the rimonabant in it [6]. Supplementing the formulation with an MCT oil did not affect the loading capacity or the encapsulation efficacy. However, the time until the formation of the second sediment was later compared to formulations without MCT oil. For example, crystal formation appeared 2 days after preparing the ester-terminated 100 kDa PLGA NPs without MCT oil. It took almost 10 days for the crystals to appear in the same formulation containing the MCT oil.
4. *Solutol concentration - Effect on the free drug in the suspension.* In all the above-mentioned preparations, a high percentage of free drug remained in the suspension, not in the NPs (~4 mg per 15 mg). The reason for the increased free drug in the suspension was probably due to the high concentration of the surfactant Solutol, which resulted in dissolving the drug into Solutol micelles. Therefore, the concentration of Solutol was gradually reduced and the encapsulation efficacy was determined. Indeed, decreasing the percentage of Solutol to even 0.1% of the final concentration resulted in a very small amount of free drug in the suspension (~200 µg per 15 mg), with no effect on the sediment formation.

   The Cryo-TEM images of the initial formulation (5 mg rimonabant, 90 mg acid-terminated 16 kDa PLGA in 0.5% Solutol) and one of the final optimized formulations (15 mg rimonabant, 65 mg ester-terminated 70 kDa PLGA, 20 mg OCA, and 30 mg MCT oil in 0.1% Solutol) are presented in **Supplementary Figure 1**.

Supplementary Figure 1. Cryo-TEM images of the initial formulation (**A**; 5 mg rimonabant, 95 mg 16 kDa PLGA) and one of the final optimized formulations (**B**; 15 mg rimonabant, 65 mg 70 kDa PLGA, 20 mg OCA, and 30 mg MCT oil).

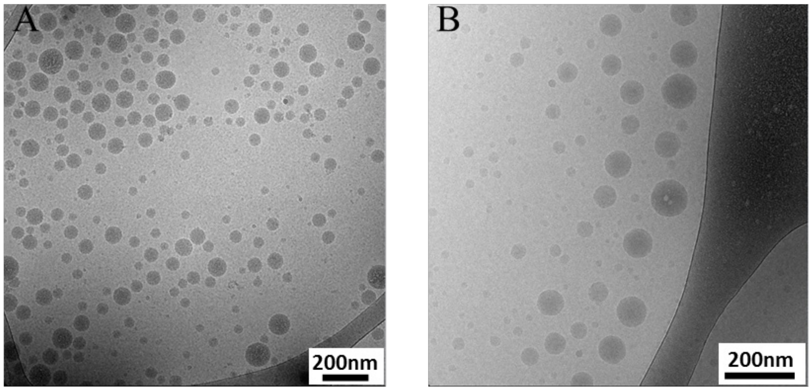

Following the optimization process described above, different formulations (**Supplementary Table 2**) were chosen to be iv administered to C57bl/6Jhsd mice, and the brain and serum levels of rimonabant were determined. The formulations were freshly prepared before injection. One hour following administration, the mice were anesthetized, blood was collected, and the mice were perfused with 1xPBS for 1 min to remove the drug from the intravascular space before removing the brain. Brain and serum were extracted, and the drug levels were determined by LC-MS/MS. As shown in **Supplementary Figure 2**, no differences in the penetration of the drug into the brain were found in comparison to the levels of free drug administered at the same dose.

Supplementary Table 2. Formulations prepared by nanoprecipitation for *in vivo* administration

No.	Ingredients	Size (d.nm)	PDI	Zeta (mV)	Encapsulation Efficacy (%)	Loading capacity (%)
1	28.75 mg E/T 37 kDa PLGA<br>10 mg OCA<br>7.5 mg Rimonabant<br>3.75 mg MCT oil	119.3	0.074	-37.7	83	11.0
2	28.75 mg E/T 75 kDa PLGA 75:25<br>10 mg OCA<br>7.5 mg Rimonabant<br>3.75 mg MCT oil	128.7	0.102	-35.7	68	9.55
3	28.75 mg E/T 70 kDa PLGA<br>10 mg OCA<br>7.5 mg Rimonabant<br>3.75 mg MCT oil	112.8	0.068	-39.5	76.88	10.65
4	28.75 mg E/T 37 kDa PLA<br>10 mg OCA<br>7.5 mg Rimonabant<br>3.75 mg MCT oil	133.3	0.077	-39.7	70.46	9.44
5	28.75 mg free acid/T 16 kDa PLGA<br>10 mg OCA<br>7.5 mg Rimonabant<br>3.75 mg MCT oil	95.22	0.076	-40.6	61.8	8.6

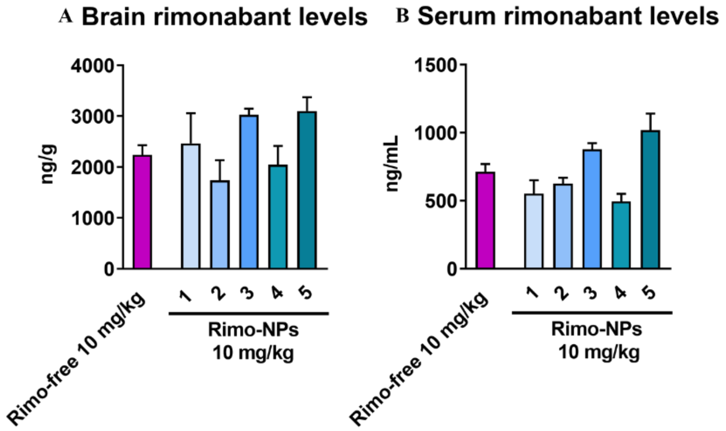

Supplementary Figure 2. Similar brain and circulating levels of rimonabant injected (at a dose of 10 mg/kg, iv) either as a free solution or encapsulated in different NP formulations, as described in **Supplementary Table 2**. Data presented represent the mean ± SEM of 3-8 animals per group.

Emulsion Evaporation

The second approach used to encapsulate rimonabant in NPs was the Emulsion Evaporation method.

Single Emulsion Evaporation

For a typical formulation using the single-emulsion evaporation technique, a solution of 10 mg rimonabant in 200 µL EtOH was added to 3 mL DCM or EtOAc containing 3% PLGA. The solution was added dropwise to 2% PVA in 1xPBS buffer solution (in 25 mL/10 mL for DCM/EtOAc, respectively, 1xPBS buffer) and then sonicated with a microtip probe sonicator (Vibra-Cell tip sonicator, Sonic&Materials, Inc., CT, USA), for 105 s at 80% amplitude over an ice bath, to form a single emulsion (O/W). DCM or EtOAc was eliminated by evaporation under reduced pressure using a rotary evaporator (Buchi, Switzerland), resulting in the formation of NPs. The NPs were recovered by ultracentrifugation (20,000 rpm, for 20 min, at 4°C), washed twice

(with a 10% albumin solution and then with 1xPBS) to remove PVA and unencapsulated rimonabant. The pellet was resuspended in 10% sucrose solution and lyophilized. Dry lyophilized NPs were stored at -20 °C until use.

Double Emulsion Evaporation

HSA (CSL Behring) solution (2%) was added dropwise (1 mL or 300 µL) under ultra-sonication to a 3 mL EtOAc or DCM solution (respectively) containing 10 mg rimonabant and 90 mg PLGA, and it was sonicated for 105 s at 80% amplitude over an ice bath to form a single emulsion (W/O). Then, the resulting W/O primary emulsions were added dropwise to a 10 mL or 25 mL 2% PVA solution, respectively, and sonicated again for 105 s at 80% amplitude over an ice bath, to form a double emulsion (W/O/W). Next, the organic solvent was eliminated by evaporation under reduced pressure using a rotary evaporator (Buchi, Switzerland), resulting in the formation of NPs [7].

Compared to the nanoprecipitation technique, the single-emulsion evaporation method using rimonabant-base, 16 kDa acid-terminated PLGA and EtOAc resulted in more stable NPs. However, they were too small (~100 d.nm, PDI 0.19, and zeta -5.44 mV), the content of rimonabant was relatively low (~6 mg per 10 mg added), and there was a significant amount of free drug in the suspension (>1 mg). Therefore, an attempt was made to encapsulate rimonabant-base in NPs by using the double-emulsion evaporation technique with the same polymer in which the inner phase contained a 2% solution of albumin as a chelator of rimonabant, because rimonabant has a high serum protein binding [8] (>98%), and albumin is a serum protein that exists at 5% in serum. Indeed, the results were promising, demonstrating that only 250 µg rimonabant were found in the external solution, and that larger NPs were obtained (~220 nm d.nm). The disadvantage was a very high PDI (0.3-0.5). Replacing EtOAc with DCM and using the single

emulsion evaporation technique with rimonabant-base and 16 kDa acid-terminated PLGA resulted in an improvement in the formulation in terms of particle size (263.7 d.nm), PDI (0.265), encapsulation efficacy (~8 mg per 10 mg rimonabant added), and a negligible amount of free drug in the suspension. Moreover, using the double-emulsion evaporation technique with DCM resulted in a narrow range of PDI (0.26 with DCM vs. 0.4 with EtOAc) and a high rimonabant content (8 mg with DCM vs. 6 mg with EtOAc). In attempting to find an explanation for the rapid release of rimonabant, we performed a release test for the two preparations described above (16 kDa acid-terminated PLGA and DCM as a solvent) in 50% human serum in a 1x PBS solution, before and after NPs were washed with a 10% human albumin solution in 1x PBS. The latter step was done to release the drug loosely bound to the surface of the NPs. As shown in **Supplementary Figure 3A, C**, between 20% and 40% of the drug was rapidly released from the NPs immediately upon exposure to a 50% serum solution. When the NPs were first washed with a 10% albumin solution, most of the rimonabant was released to the albumin solution, leaving a lower amount of the drug in the NPs, which remained encapsulated (**Supplementary Figure 3B, D**). Data regarding the amount of rimonabant before and after washing the formulation with a 10% albumin solution are presented in **Supplementary Table 3**.

Supplementary Figure 3. Release of rimonabant base from PLGA 16kDa acid-terminated NPs, prepared using a single- (**A, B**) or double- (**C, D**) emulsion evaporation technique. The release test was performed in 50% human serum at 4°C. Measurements were taken before (**A, C**) and after (**B, D**) the preparation was washed with a 10% albumin solution. Data are presented as the mean ± SEM of 2 samples at each time point.

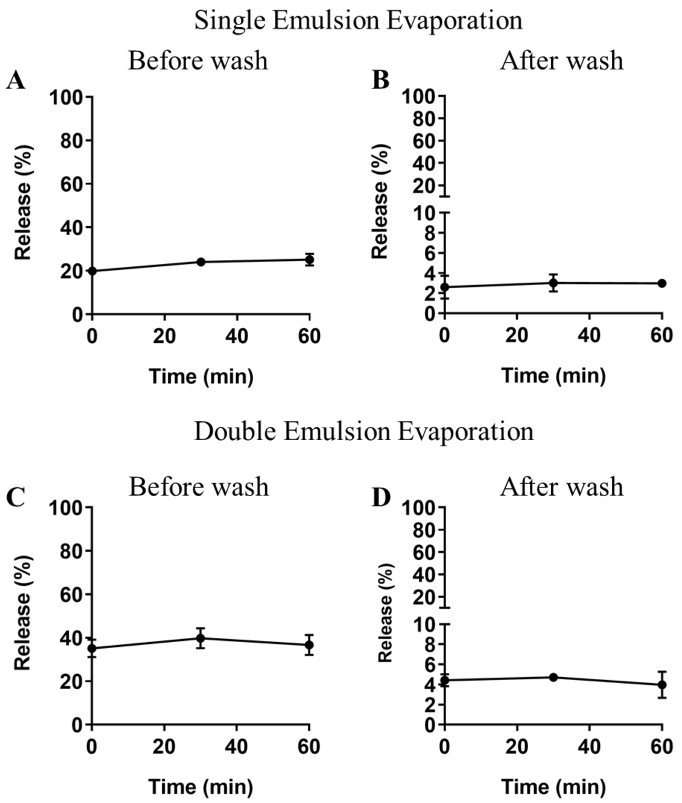

	Single-emulsion evaporation	Double-emulsion evaporation
**Before wash**	8,547.5±190.5	8,379±469.3
**After wash**	3,230±193	2,707.32±292.35

Supplementary Table 3. Amounts of rimonabant-base (µg) encapsulated in NPs before and after washing the NPs with a 10% albumin solution.

To determine whether the rapid release of rimonabant from the NPs was due to the MW of polymer consisting of the NPs, a new formulation with a different polymer was prepared using the single-emulsion evaporation technique. The polymer chosen was a 100 kDa ester-terminated PLGA

LA:GA (50:50), which for single emulsion, resulted in an NP size of 239.5 d.nm, PDI 0.12, an encapsulation efficacy of 67.6%, and a loading capacity of 7.63%. For double emulsion, the encapsulation efficacy was 33.2% and the loading capacity was 3.5%. However, a very rapid release of the drug from the nanoparticles was still observed (**Supplementary Figure 4**). Data regarding the amount of rimonabant in the NPs measured before and after washing the NPs prepared with a 10% albumin solution are presented in **Supplementary Table 4**.

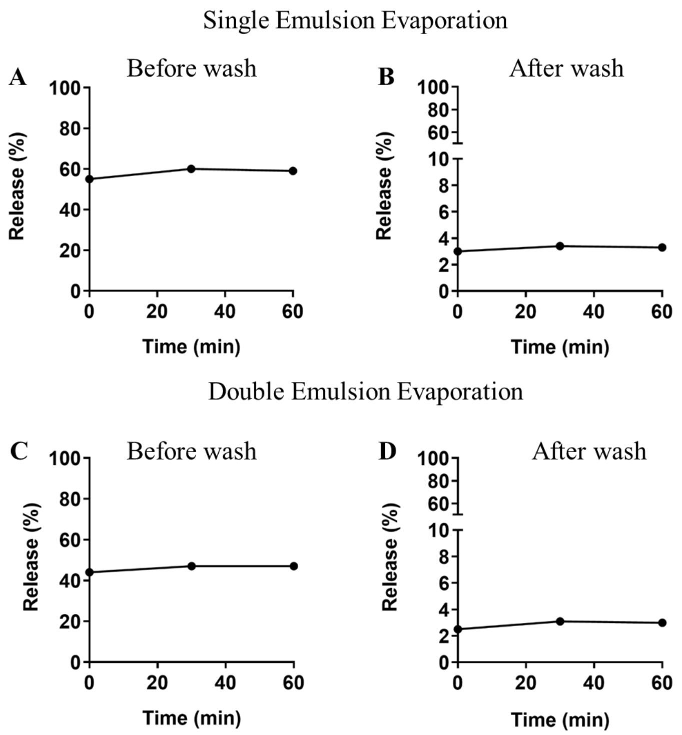

Supplementary Figure 4. Release of rimonabant-base from 100 kDa ester-terminated PLGA NPs prepared using a single- (**A, B**) or double- (**C, D**) emulsion evaporation technique. The release test was performed in 50% human serum at 4°C. Measurements were made before (**A, C**) and after (**B, D**) the NP preparation was washed with a 10% albumin solution. Data are presented as the value of 1 sample at each time point.

	**Single emulsion evaporation**	**Double emulsion evaporation**
**Before washing**	7,206	3,398
**After washing**	1,098	599

Supplementary Table 4. Amounts of rimonabant-base (µg) measured in NPs before and after washing the NPs with a 10% albumin solution.

Examination of the release kinetics and the content of rimonabant as HCl, in the NPs (before and after washing the NPs with a 10% albumin solution) revealed no significant improvement in the release kinetics of rimonabant from NPs. However, a much higher amount of rimonabant remained in the NPs after the washing step (for single-emulsion evaporation: 3,156 µg with rimonabant-HCl vs. 1,098 µg with rimonabant-base; for double-emulsion evaporation: 2,343 µg with rimonabant-HCl vs. 599 µg with rimonabant-base, as presented in **Supplementary Figure 5 and Supplementary Table 5**.

Supplementary Figure 5. Release of rimonabant-HCl from 100 kDa ester-terminated PLGA NPs prepared using a single- (**A, B**) or double- (**C, D**) emulsion evaporation technique. The release test was performed in 50% human serum at 4°C. Measurements were made both before (**A, C**) and after (**B, D**) the preparation was washed with a 10% albumin solution. Data are presented as values of 1 sample at each time point.

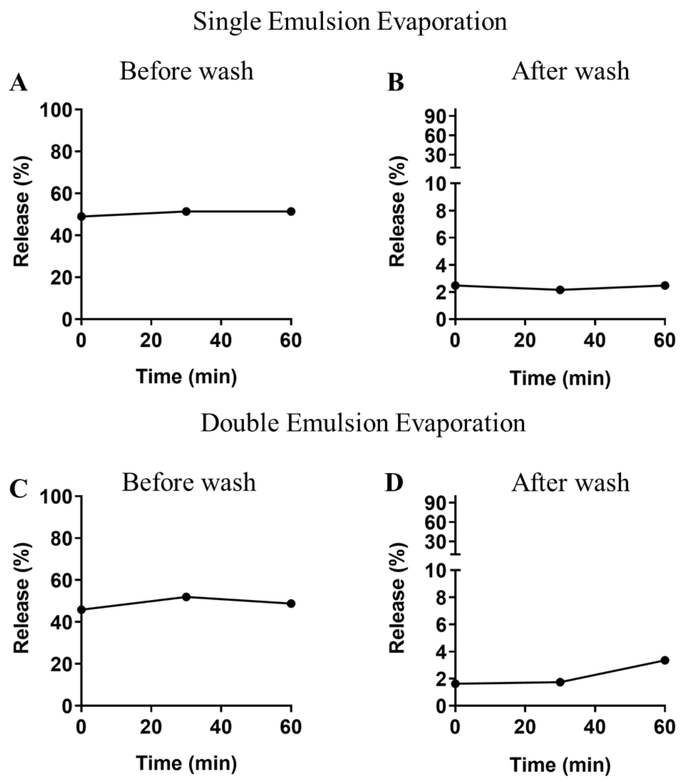

	**Single-emulsion evaporation**	**Double-emulsion evaporation**
**Before washing**	8,228	7,650
**After washing**	3,156	2,343

Supplementary Table 5. Amount of rimonabant-HCl (μg) encapsulated in PLGA 100 kDa ester-terminated NPs before and after washing the NPs with a 10% albumin solution.

To determine whether acid-terminated PLGA of a higher MW would reduce the rapid release of rimonabant-base from the NPs, a new formulation with a different polymer was prepared using the single- and double-emulsion evaporation techniques. The polymer chosen was an acid-terminated 50 kDa PLGA LA:GA (50:50). Indeed, significant improvements in the release kinetics and in the amount of rimonabant remaining in the NPs were achieved (**Supplementary Figure 6 and Supplementary Table 6**), suggesting that 50 kDa acid-terminated PLGA is the preferred polymer to use.

Supplementary Figure 6. Release test of rimonabant-base from 50 kDa acid-terminated PLGA NPs prepared using the single- (**A, B**) or double- (**C, D**) emulsion evaporation technique. The release test was performed in 50% human serum at 4°C. Measurements were made before (**A, C**) and after (**B, D**) the preparation was washed with a 10% albumin solution. Data are presented as the mean ± SEM of 1-2 samples at each time point.

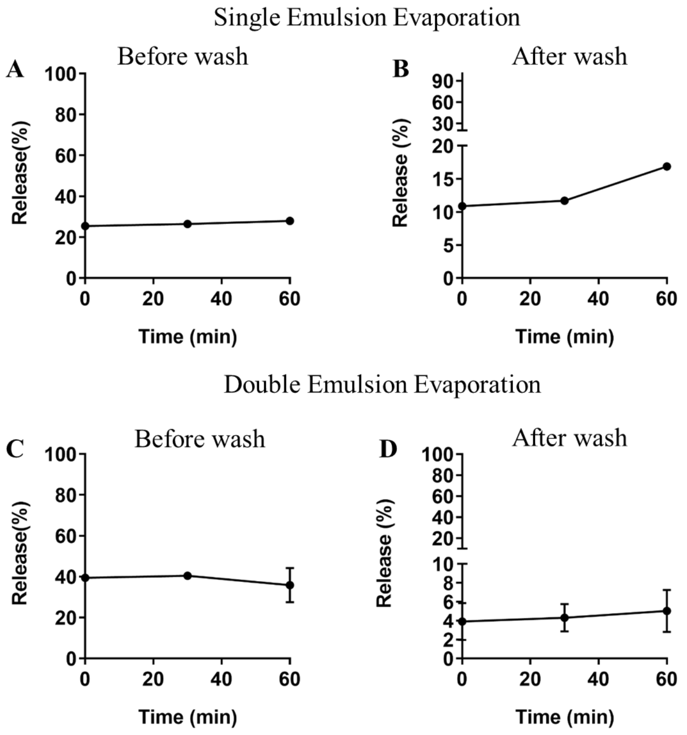

	Single-emulsion evaporation	Double-emulsion evaporation
**Before washing**	9,264	9,893±188.7
**After washing**	6,342	4,032±268

Supplementary Table 6. Amounts of rimonabant-base (µg) encapsulated in PLGA 50 kDa acid-terminated NPs before and after washing the NPs with a 10% albumin solution.

Next, the influence of rimonabant in its acid form (HCl) on its content in NPs and its release kinetics from NPs was examined before and after washing the formulation with a 10% albumin solution. It was found to provide no added advantage over the rimonabant-base (**Supplementary Figure 7 and Supplementary Table 7**).

Supplementary Figure 7. Release of rimonabant-HCl from 50 kDa acid-terminated PLGA NPs prepared using single- (**A, B**) or double- (**C, D**) emulsion evaporation techniques. The release test was performed in 50% human serum at 4°C. Measurements were made both before (**A, C**) and after (**B, D**) the preparation was washed with a 10% albumin solution. Data are presented as the mean ± SEM of 1-3 samples at each time point.

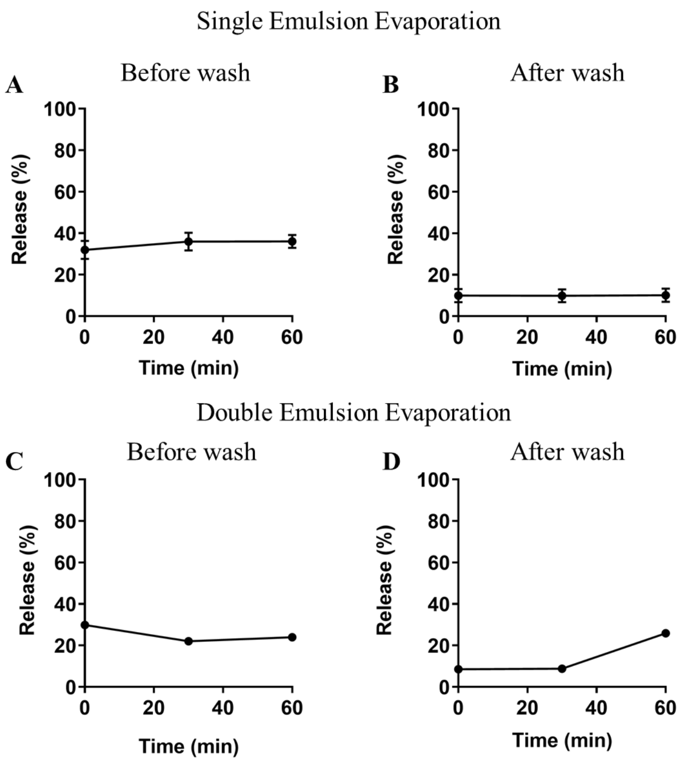

	**Single-emulsion evaporation**	**Double-emulsion evaporation**
**Before washing**	9,505.67±411.49	8,035
**After washing**	4,725.03±851.6	4,770

Supplementary Table 7. Amounts of rimonabant-HCl (µg) encapsulated in PLGA 50 kDa acid-terminated NPs before and after washing the NPs with a 10% albumin solution.

The screening tests described above demonstrated that no significant difference exists between single- and double-emulsion evaporation techniques in terms of drug encapsulation efficacy and release kinetics. The preferred polymer for encapsulating rimonabant, as assessed in terms of drug release, and the amount of the drug in NPs after an albumin wash, is 50 kDa acid-terminated PLGA. Moreover, the form of rimonabant should be acidic (HCl).

Liposome preparation

POPC(palmitoyl-2-oleoyl-sn-glycero-3-phosphocpalmitoyl-2-oleoyl-sn-glycero-3-phosphocholine) (110 mg), cholesterol (5 mg), PG (egg phosphatidylglycerol) (30 mg), and rimonabant (3 mg) were solubilized in 250 µL EtOH, 250 µL ethyl acetate, and 500 µL 10% sucrose. The solution was heated to 40 °C and lyophilized. Next, the powder was resuspended in water and extruded through polycarbonate filters with defined sizes. The lipids were extruded ten times for 130-140 nm size through filters of 400 nm and 100 nm. The different changes between the formulations are the amount of POPC in comparison to cholesterol, and the inner water phase with or without albumin or iron. The size was between 100 and 600 nm, depending on the shape of the liposomes: regular or multilamellar (**Supplementary Table 8**). The multilamellar liposomes were prepared in order to reduce the release of rimonabant from liposomes. The release of rimonabant was very low in 50% FCS in PBS, but when tested on 50% human serum in PBS, the release rate was increased.

Supplementary Table 8. Liposomal formulations.

No.	Ingredients	Size (d.nm)	Loading capacity (%)
1	POPC liposomes	130	
2	POPC liposomes (*1.5 POPC)	130 or 600	
3	POPC liposome within Lipidmix (HSPC)	110	17.6
4	POPC liposome (x1.5) within Lipidmix	90	11
5	POPC liposome (90 nm) within (HSPC/cholesterol)	600	39.1

Supplementary Figure 8. Cryo-TEM images of the liposomes. Unilamellar liposome formulation (**A**), and multilamellar liposome formulation (**B**).

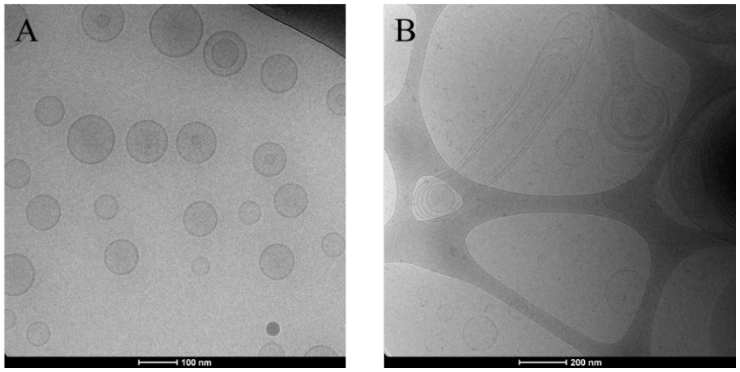

After the optimization process with different ratios of POPC vs. cholesterol, single vs. multilamellar liposomes, and with albumin or iron in the aqua core, the different liposomal formulations were injected iv into male C57Bl/6 mice, and the brain and serum levels of rimonabant were determined by LC-MS/MS. As shown in **Supplementary Figure 9**, no differences in brain penetration of the drug were found, compared to levels of free drug administered at the same dose.

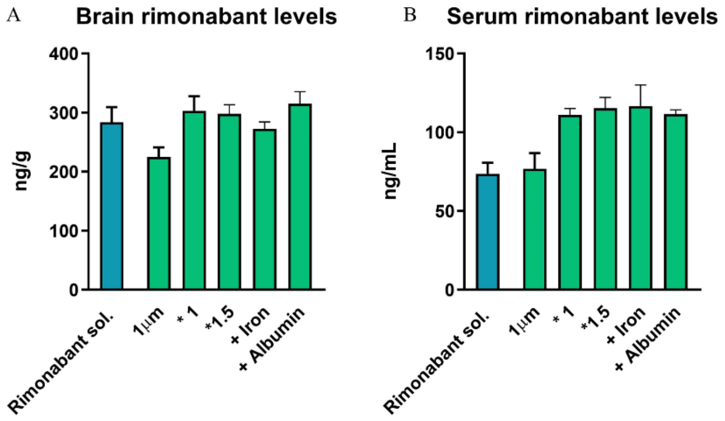

Supplementary Figure 9. Brain and circulating levels of rimonabant (liposomes). Similar brain (**A**) and circulating (**B**) levels of rimonabant injected (at a dose of 3 mg/kg, iv) either as a free solution or encapsulated in different liposome formulations as described in **Supplementary Table 8**. Data represent the mean ± SEM of 3 animals per group.

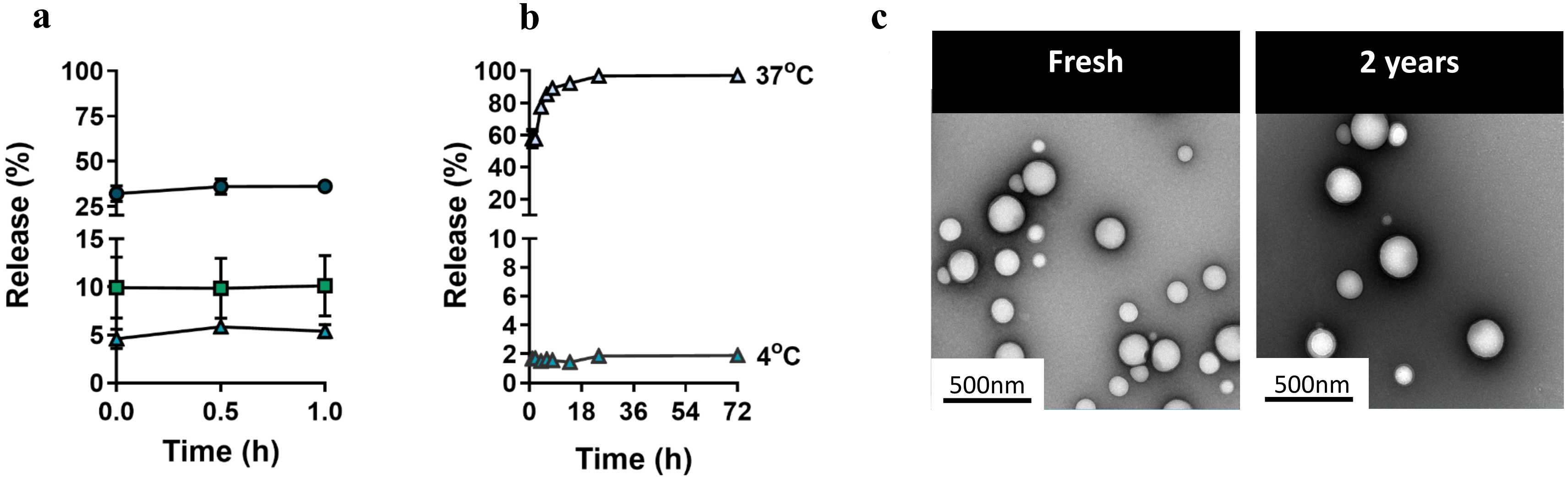

Supplementary Figure 10. Stability assessment of Rimo-NPs. (**a**) The leakage of rimonabant from NPs stored at 4°C in 50% human plasma in PBS, before and after washing Rimo-NPs with 10% albumin solution, upper line (circles) and the middle line (squares), respectively, and after washing and reconstituting the lyophilized powder, the lower line (triangles). (**b**) The effect of temperature on the release kinetics of rimonabant from washed and reconstituted Rimo-NPs. (**c**) Transmission electron microscopy (TEM) micrographs of Rimo-NPs depicting the unaffected spherical shape of the lyophilized NPs under storage (Uranyl acetate negative staining, magnification 25 K, scale bar = 500 nm). Data represent the mean ± SEM from 3 independent formulations per condition.

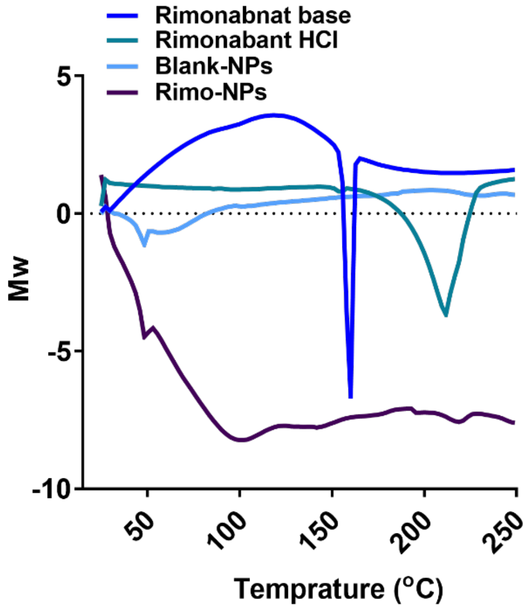

Supplementary Figure 11. DSC thermogram of rimonabant base, rimonabant HCl, Blank-NPs, and Rimo-NPs.

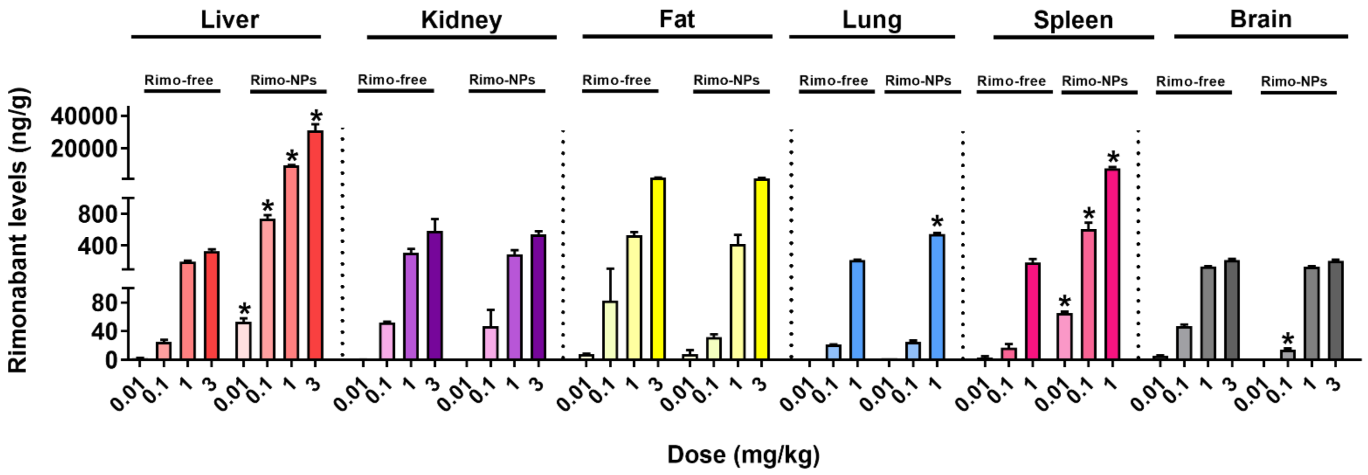

Supplementary Figure 12. Rimonabant levels in the liver, kidney, fat, lung, spleen, and brain 1 h after of iv administration of free rimonabant and Rimo-NPs at 0.01-3.0 mg/kg. Data represent the mean ± SEM from 3 mice per group. *p<0.05 relative to free rimonabant levels at the respective dose.

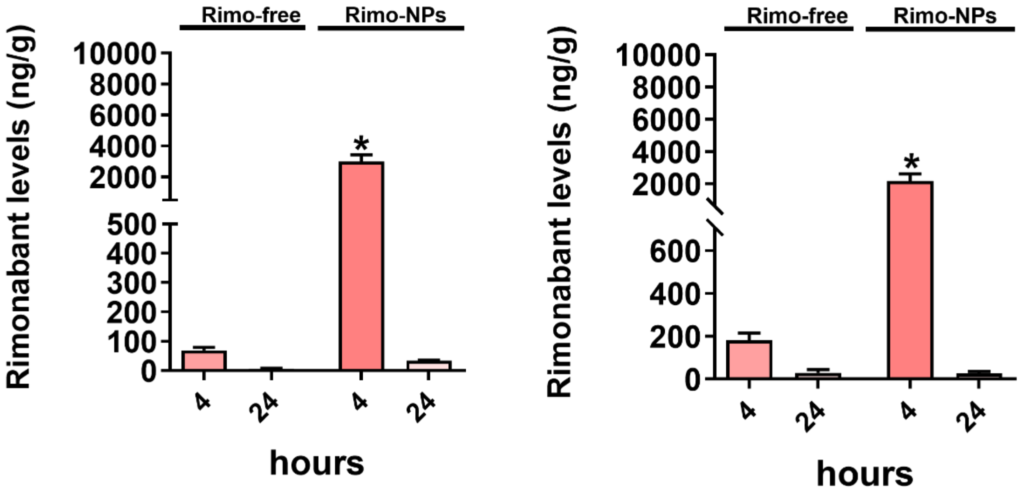

Supplementary Figure 13. Rimonabant levels in the liver 4- and 24-hours following iv (left panel) or ip (right panel) administration of free rimonabant (Rimo-free) and Rimo-NPs at a dose of 1 mg/kg. Data are presents as mean ± SEM of 3 mice per group. *p<0.05 relative to levels at the respective time after administration of free rimonabant.

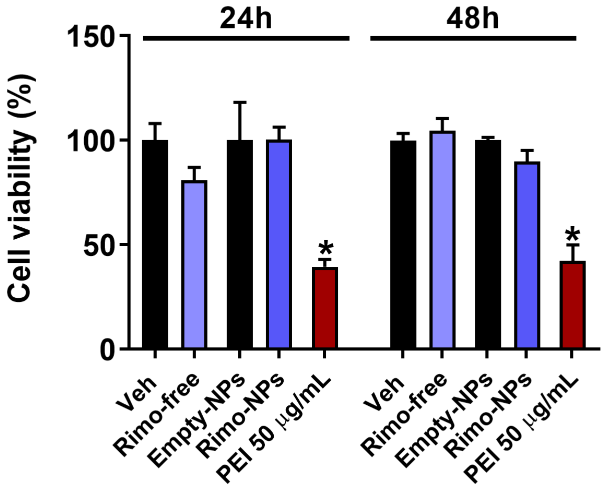

Supplementary Figure 14. Toxicity of Rimo-NPs. Cell viability measured by incubating primary hepatocytes with free rimonabant, empty NPs, and Rimo-NPs for 24-48 h. Cell viability was normalized to vehicle-treated cells. Data represent the mean ± SEM from three independent experiments. Polyethyleneimine (PEI) was used as a positive control. **p*<0.05 relative to vehicle-treated cells.

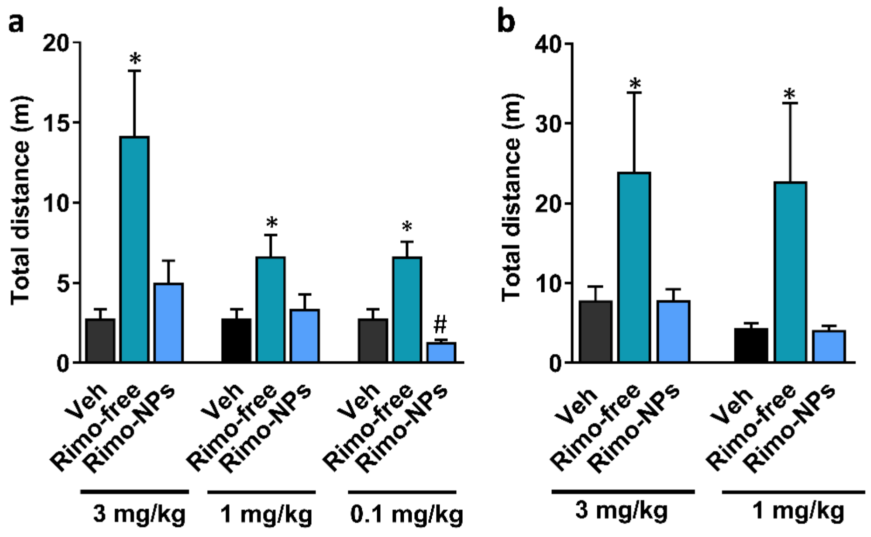

Supplementary Figure 15. Inability of Rimo-NPs to induce CNS-mediated hyperactivity. Free rimonabant, but not Rimo-NPs increased the ambulatory activity after both iv (**a**; 0.1, 1, and 3 mg/kg) and ip (**b**; 1 and 3 mg/kg) administrations. Hyperactivity was measured by counting the total distance (in meters) the mice traveled in the cage for 4 hours post-injection. Data represent the mean ± SEM from 4 mice per group. *p<0.05 relative to vehicle (Veh; free PLGA-NPs), #p<0.05 relative to free rimonabant.

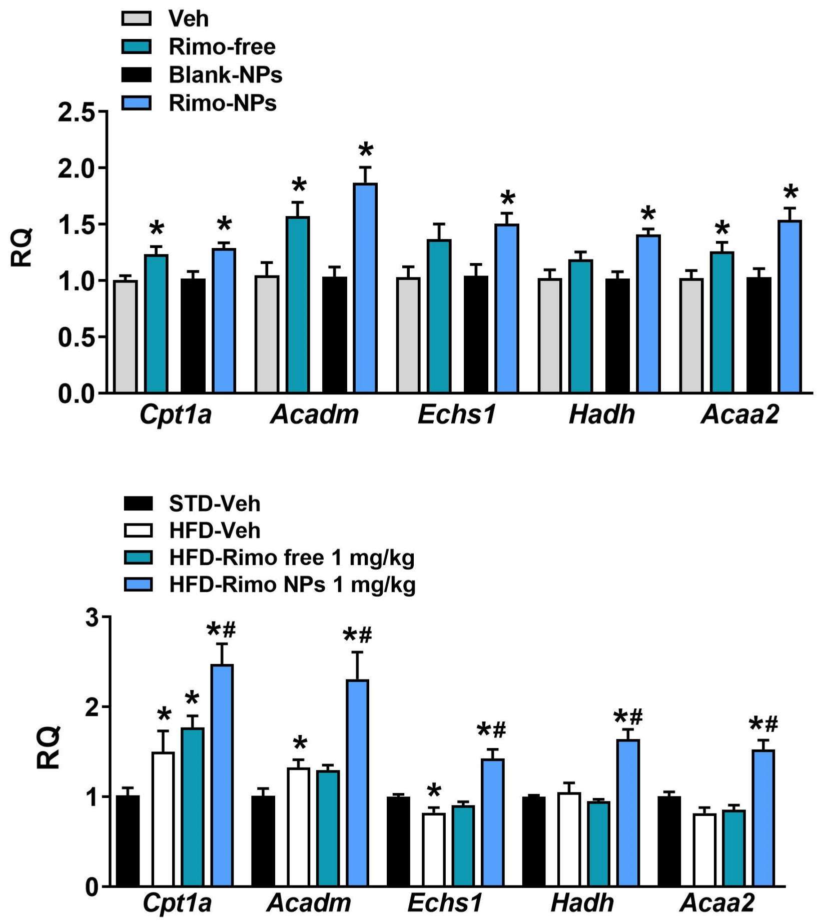

Supplementary Figure 16. Rimo-NPs enhances hepatic fatty acid utilization/oxidation. Upper Panel: Rimo-NPs affected the gene expression profile of hepatocytes exposed to lipotoxic conditions. Primary mouse hepatocytes were exposed to 0.5 mM mixture of oleate and palmitate (O:P 2:1, respectively) in the absence/presence of Rimo-free or Rimo-NPs (1 µM, each) for 24 h. Then, mRNA was extracted from the cells and qPCR was performed to assess the expression of several genes associated with fatty-acid β-oxidation. *p<0.05 relative to the corresponding vehicle (Veh)-treated cells. Lower Panel: Mice on STD or HFD for 14 weeks were treated ip with vehicle (Veh; free PLGA-NPs) or 1 mg/kg/d free rimonabant (Rimo-free) or Rimo-NPs for 28 days. Changes in mRNA levels of fatty acid β-oxidation genes. Data are presented as mean ± SEM of 7 mice per group, *p<0.05 relative to STD-Veh, #p<0.05 relative to HFD-Veh.

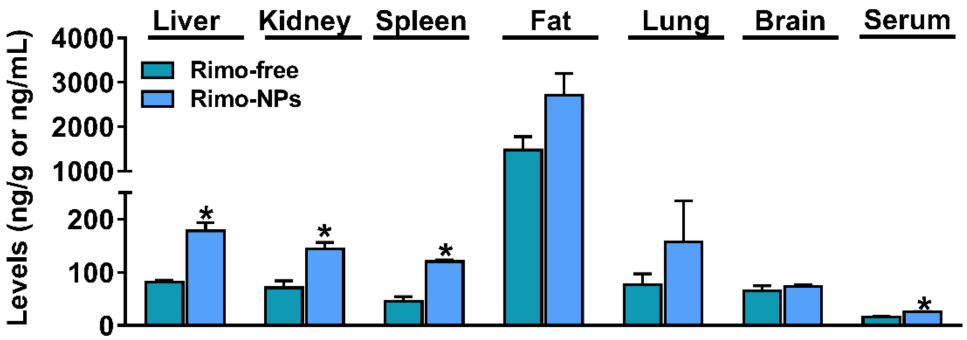

Supplementary Figure 17. Rimo-NP biodistribution in diet-induced obese mice. The accumulation of rimonabant in organs was evaluated by analyzing the rimonabant levels in the liver, kidney, spleen, fat, lung, brain, and serum 18 h post chronic 28-day injection (1 mg/kg, ip) to high-fat diet-induced obese mice. Data represent the mean ± SEM of 3 mice per group. *p<0.05 relative to free rimonabant levels in the same tissue.

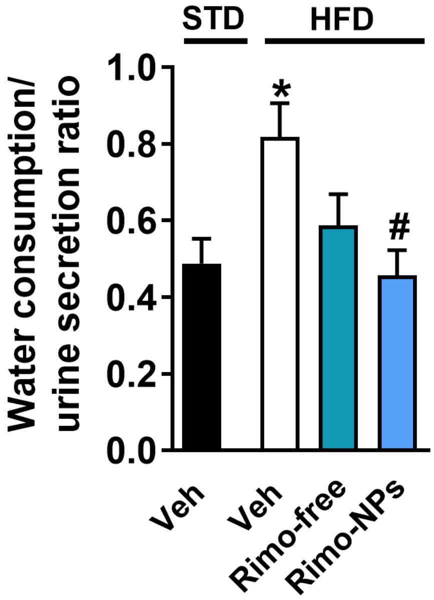

Supplementary Figure 18. Water consumption-to-urine secretion ratio following chronic treatment with Rimo-NPs. Mice on a standard diet (STD) or high-fat diet (HFD) for 14 weeks were treated with vehicle (Veh; free PLGA-NPs) or 1 mg/kg/d, ip, of free rimonabant or Rimo-NPs for 28 days. Rimo-NPs reduced the HFD-induced elevation in the water consumption-to-urine secretion ratio. Data represent the mean ± SEM from 7 mice per group. *$p<0.05$ relative to STD-Veh, #$p<0.05$ relative to HFD-Veh.

Supplementary Table 9. Mouse Primers used for RT-PCR Analysis

Gene	Forward primer (5'-3')	Reverse primer (5'-3')
Cpt1a	CCGTGAGGAACTCAAACCTATT	CAGGGATGCGGGAAGTATTG
Acadm	CAGCCAATGATGTGTGCTTAC	CATACTCGTCACCCTTCTTCTC
Echs1	GGACTGTTACTCCAGCAAGTTC	CCCACCAAGAGCATAACCATT
Hadh	CCAAGAAGGGAATTGAGGAGAG	ACAAACTCATCTCCAGCCTTAG
Acaa2	CAGAGGTGGAAAGCTGCTAA	GCATGGTCTGTTTGCCTTTC
Actb	GGCTGTATTCCCCTCCATCG	CCAGTTGGTAACAATGCCATGT

Cpt1a, Carnitine palmitoyltransferase 1A; *Acadm*, acyl-CoA dehydrogenase medium chain; *Echs1*, enoyl-CoA hydratase, short chain 1; *Hadh*, hydroxyacyl-CoA dehydrogenase; *Acaa*, acetyl-CoA acyltransferase; *Actb*, beta actin.

References:

1. Raemdonck, K., et al., *Merging the best of both worlds: hybrid lipid-enveloped matrix nanocomposites in drug delivery.* Chem Soc Rev, 2014. **43**(1): p. 444-72.
2. Fessi, H., et al., *Nanocapsule formation by interfacial polymer deposition following solvent displacement.* International Journal of Pharmaceutics, 1989. **55**(1): p. R1-R4.
3. Karra, N., et al., *Antibody conjugated PLGA nanoparticles for targeted delivery of paclitaxel palmitate: efficacy and biofate in a lung cancer mouse model.* Small, 2013. **9**(24): p. 4221-36.
4. Martín-Banderas, L., et al., *Cannabinoid derivate-loaded PLGA nanocarriers for oral administration: formulation, characterization, and cytotoxicity studies.* Int J Nanomedicine, 2012. **7**: p. 5793-806.
5. Danhier, F., et al., *PLGA-based nanoparticles: an overview of biomedical applications.* J Control Release, 2012. **161**(2): p. 505-22.
6. Badihi, A., et al., *Topical nano-encapsulated cyclosporine formulation for atopic dermatitis treatment.* Nanomedicine, 2020. **24**: p. 102140.
7. Cohen-Sela, E., et al., *A new double emulsion solvent diffusion technique for encapsulating hydrophilic molecules in PLGA nanoparticles.* Journal of Controlled Release, 2009. **133**(2): p. 90-95.
8. Xie, S., et al., *The endocannabinoid system and rimonabant: a new drug with a novel mechanism of action involving cannabinoid CB1 receptor antagonism--or inverse agonism--as potential obesity treatment and other therapeutic use.* J Clin Pharm Ther, 2007. **32**(3): p. 209-31.
